# Recent progress in understanding ferroptosis mechanisms in infectious diseases

**DOI:** 10.3389/fcimb.2026.1778721

**Published:** 2026-03-30

**Authors:** Fei Li, Xiangpeng Chen

**Affiliations:** 1 Laboratory of Infection and Virology, Beijing Pediatric Research Institute, Beijing Children’s Hospital, Capital Medical University, National Center for Children’s Health, Beijing, China; 2 Beijing Key Laboratory of Core Technologies for the Prevention and Treatment of Emerging Infectious Diseases in Children, Key Laboratory of Major Diseases in Children (Ministry of Education), Research Unit of Critical Infection in Children (Chinese Academy of Medical Sciences), Beijing, China

**Keywords:** ferroptosis, iron metabolism, lipid peroxidation, pathogen infection, therapeutic strategies

## Abstract

Ferroptosis, characterized by lipid peroxidation and iron-dependent oxidative damage, is a crucial factor in various diseases. Although researchers have extensively characterized ferroptosis in cancer and neurodegenerative disorders, its interaction with pathogenic infections remains underexplored. Recent research indicates that ferroptosis contributes to host cell damage during pathogen invasions, impacting disease outcomes. This review summarizes the characteristics, mechanisms, and regulatory networks of ferroptosis. It delineates the key regulatory steps of ferroptosis during infections caused by various pathogens, including viruses, bacteria, fungi, and parasites. Additionally, it examines changes in host markers and related signaling pathways. Furthermore, this review explores the potential similarities and differences among these pathogens and discusses therapeutic strategies for addressing pathogen-related diseases through ferroptosis-dependent mechanisms.

## 1 Introduction

Ferroptosis, an identified form of regulated cell death, has garnered significant attention due to its distinctive molecular mechanisms and implications in various diseases. Unlike conventional cell death pathways such as necrosis and apoptosis, ferroptosis has distinctive features. It is characterized by iron-dependent lipid peroxidation and the accumulation of reactive oxygen species (ROS) (Dixon et al., 2012; Zheng and Conrad, 2025). While its molecular mechanisms remain incompletely understood, ferroptosis plays a critical role in disease pathogenesis, including cancer, neurodegenerative disorders, and ischemia-reperfusion injury (Dixon et al., 2012).

Despite substantial progress in elucidating the role of ferroptosis in disease, its interplay with pathogen infections has been relatively understudied. However, emerging evidence indicates a critical relationship between ferroptosis and host cell damage during pathogen invasions. Pathogens and the host immune response collectively dysregulate intracellular iron metabolism and ROS levels, ultimately leading to membrane damage via lipid peroxidation (Amaral and Namasivayam, 2021). This intricate relationship highlights ferroptosis as a key mechanism in infection-related pathogenesis.

In this review, we present a comprehensive overview of ferroptosis in pathogen-related infections, discussing its implications for disease progression and treatment. Specifically, we examine the similarities and differences in ferroptosis mechanisms across various pathogens, highlighting how these distinctions can inform future therapeutic strategies and related clinical challenges. Ultimately, we aim to provide insights into the therapeutic potential of ferroptosis-targeted strategies in infectious disease management.

## 2 Overview of ferroptosis

Ferroptosis, first identified in 2003 and formally named in 2012, is a distinct form of regulated cell death characterized by unique morphological, biochemical, and genetic features (Dixon et al., 2012; Dolma et al., 2003). Morphologically, ferroptosis presents with mitochondrial abnormalities, including shrinkage, increased membrane density, cristae loss, and plasma membrane rupture. Biochemically, ferroptosis depends on two key processes. ROS accumulation occurs primarily through ferrous iron (Fe^2+^)-mediated Fenton reactions. Additionally, enzymatic peroxidation of membrane polyunsaturated fatty acids (PUFAs) produces cytotoxic lipid peroxides. Together, these processes cause progressive membrane damage. Genetically, ferroptosis is associated with altered expression of key genes, such as glutathione peroxidase 4 (GPX4), acyl-CoA synthetase long-chain family member 4 (ACSL4), and prostaglandin-endoperoxide synthase 2 (PTGS2). However, definitive molecular markers for ferroptosis remain elusive (Shen et al., 2025; Stockwell, 2022; Tang et al., 2021; Xiao et al., 2025a; Xiao et al., 2025a; Xiao et al., 2025b).

Cells employ various mechanisms to counter excessive lipid peroxides and prevent ferroptosis. Four main pathways have been identified:

The SLC7A11-GPX4 pathway: Active in both cytoplasm and mitochondria, this pathway relies on GPX4 to maintain cellular redox balance (Huang et al., 2025). Inhibition of GPX4 reduces intracellular glutathione (GSH) levels, disrupting redox balance and inducing lipid peroxide accumulation, thus triggering ferroptosis. Classic ferroptosis inducers like Erastin inhibit the amino acid transporter solute carrier family 7 member 11 (SLC7A11), thereby suppressing GPX4 levels. In addition, (1S,3R)-RSL3 (RSL3) directly inhibits GPX4, leading to decreased GSH levels. Recent studies indicate that peroxiredoxin 3 (PRDX3), a mitochondrial peroxidase, translocates to the cell membrane after peroxidation modification, potentially inducing ferroptosis by inhibiting cystine uptake (Cui et al., 2023; Yang et al., 2014). Furthermore, various pathogens have been shown to regulate ferroptosis through this pathway, including viruses, such as severe acute respiratory syndrome coronavirus 2 (SARS-CoV-2) (Liu et al., 2023b; Sun et al., 2025), hepatitis B virus (HBV) (Wang et al., 2023c), Epstein-Barr virus (EBV) (Yuan et al., 2022), rotavirus (RV), and highly pathogenic avian influenza A virus subtype H5N1 (H5N1) and pandemic influenza A virus subtype H1N1(H1N1) (Wei et al., 2024; Zhou et al., 2024a), and Japanese encephalitis virus (JEV) (Zhu et al., 2024). Additionally, bacteria including *Pseudomonas aeruginosa* (*P. aeruginosa*) (Dar et al., 2021), *Staphylococcus aureus* (*S. aureus*) (Hu et al., 2023a), and *Mycobacterium tuberculosis* (*M. tuberculosis*) (Ma et al., 2022a) modulate ferroptosis via this pathway.

The FSP1-CoQH2 antioxidant pathway: Located on the cell membrane, this pathway involves ferroptosis suppressor protein 1 (FSP1), also known as apoptosis-inducing factor mitochondria-associated 2 (AIFM2). FSP1 employs nicotinamide adenine dinucleotide phosphate (NADPH) to produce reduced coenzyme Q10 (CoQ10), which degrades lipid peroxides on the cell membrane and prevents ferroptosis (Doll et al., 2019). Under certain conditions, FSP1 can also inhibit ferroptosis by activating membrane repair mediated by the endosomal sorting complex required for transport III (ESCRT-III).

The DHODH-CoQH2 pathway: Located within the inner mitochondrial membrane, this pathway involves dihydroorotate dehydrogenase (DHODH), which reduces coenzyme Q (CoQ) to coenzyme QH2 (CoQH2) when intracellular GPX4 levels decrease. CoQH2, an antioxidant, captures ROS and thus inhibits ferroptosis (Mao et al., 2021).

The GCH1-BH4 pathway: This pathway involves tetrahydrobiopterin (BH4) biosynthesized by guanosine triphosphate (GTP) cyclohydrolase-1 (GCH1). BH4 induces lipid remodeling and selectively inhibits phospholipid consumption at the tail ends of polyunsaturated fatty acids to suppress ferroptosis.

The FSP1-CoQH2, DHODH-CoQH2, and GCH1-BH4 pathways all converge on suppressing lipid peroxidation through the production of antioxidant metabolites. Currently, no pathogen-related associations have been reported for these three pathways. Recent studies have identified membrane-bound O-acyltransferase domain-containing 1/2 (MBOAT1/2) as novel ferroptosis suppressors. Regulated by the estrogen receptor and androgen receptor, respectively, MBOAT1/2 suppress ferroptosis by modifying phospholipids, presenting a distinct regulatory mechanism independent of GPX4 or FSP1 (Liang et al., 2023).

## 3 The role of ferroptosis in diseases

**Figure 1:**
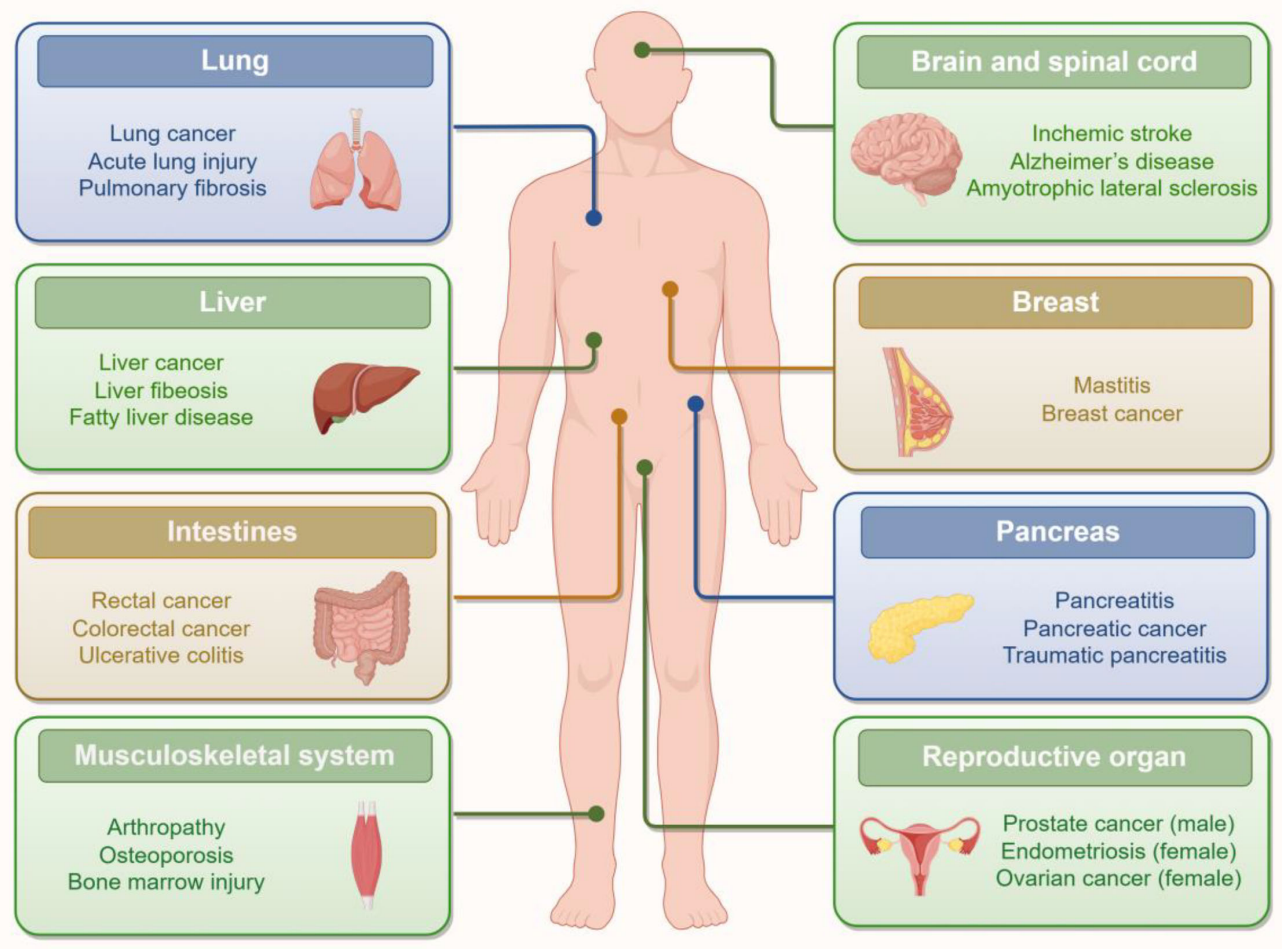
Ferroptosis in various human diseases. Ferroptosis has played important roles in multiple system diseases, such as lung diseases, nervous system diseases, breast -related diseases, liver diseases, pancreatic diseases, intestinal diseases, reproductive diseases, musculoskeletal system diseases. Created by Figdraw.

Ferroptosis plays a significant role in various diseases, particularly cancer, influencing both its occurrence and progression (**Figure 1**). Its involvement in tumor development and treatment is complex, influenced by oncogenes, tumor suppressors, and the tumor microenvironment (Xia et al., 2025; Xu et al., 2025). For example, in a K-ras-induced mouse model of lung cancer, knockout of RNA binding motif single-stranded interacting protein 1 (RBMS1) substantially suppresses lung cancer progression by inducing ferroptosis (Zhang et al., 2021). Additionally, under hypoxic conditions, hypoxia inducible factor 1 subunit alpha (HIF-1α) upregulates solute carrier family 1 member 1 (SLC1A1) to drive solid tumor resistance to ferroptosis. Concurrently, HIF-1α-driven lactate accumulation via lactate dehydrogenase A (LDHA) further enhances this resistance (Yang et al., 2023). Exploiting the high metabolic capacity of tumor cells, certain chemotherapeutic drugs like sorafenib and sulfasalazine induce ferroptosis in various cancer types. For example, melanoma cells resistant to targeted kinase inhibitors and immunotherapy become sensitive to ferroptosis, thereby enhancing the efficacy of targeted and immune therapies (Tsoi et al., 2018). In a pancreatic cancer mouse model, the small molecule N6-furfuryl adenine 11 (N6F11) selectively triggers GPX4 degradation in tumor cells, inducing ferroptosis. This process subsequently initiates high mobility group box 1 (HMGB1)-dependent anti-tumor immunity mediated by CD8^+^ T cells (Li et al., 2023a).

Beyond cancer, mounting evidence suggests that iron dysregulation, oxidative stress, and GPX4 suppression are key features of ferroptosis in neurodegenerative diseases and cognitive impairments. Ferroptosis plays a crucial role in the occurrence and progression of various neurodegenerative diseases, including Alzheimer’s disease, Parkinson’s disease, Huntington’s disease, and stroke (Dang et al., 2022). For instance, iron overload can trigger ferroptosis in microglia via the vesicle transport gene SEC24 homolog B (SEC24B), leading to neurodegenerative changes (Ryan et al., 2023).

Infectious pathogens often trigger oxidative stress responses in host cells, which can either facilitate pathogen infection or counteract it by promoting host cell death to halt infection progression. Iron ions are vital for cellular physiology and are particularly important during pathogen infections, placing iron at a critical nexus of host-pathogen interactions, where it serves as a key determinant of micronutrient competition between pathogens and hosts. To date, at least 50 pathogen-associated infections have been linked to ferroptosis.

In 2002, Barluzzi et al. reported that iron overload worsens *Cryptococcus neoformans* (*C. neoformans*)-induced meningoencephalitis. However, as the concept of ferroptosis had yet to be established, a definitive connection could not be drawn (Barluzzi et al., 2002). In July 2018, Bogacz et al. suggested that deficiency in *Trypanosoma cruzi tryparedoxin* peroxidase results in lethal iron-dependent lipid peroxidation, leading to ferroptosis, with mitochondrial iron playing a pivotal role. Later that year, in November, Dar et al. found that Pseudomonas aeruginosa induces bronchial epithelial cell ferroptosis by exploiting host polyunsaturated phospholipids (Bogacz and Krauth-Siegel, 2018; Dar et al., 2018). These studies marked the beginning of research into the relationship between ferroptosis and pathogen infection. In 2020, Kuo et al. demonstrated that emodin inhibits hepatic stellate cell activation by hepatitis B virus X protein (HBx) through endoplasmic reticulum stress and ferroptosis pathways, thereby suppressing liver fibrosis and introducing ferroptosis into virus research (Kuo et al., 2020).

## 4 The role of ferroptosis in infection

### 4.1 Ferroptosis in viral infections

The interaction between viral infection and ferroptosis is complex and multifaceted. Iron, crucial for cellular enzymes, maintains cell function and supports viral replication. Viruses can alter cellular iron metabolism by disrupting iron uptake mechanisms or by exploiting iron transport proteins as viral receptors. While virus-induced ferroptosis can limit the spread of infection within the host, some viruses have evolved to exploit this pathway to facilitate their own proliferation and evade immune surveillance. As previously described, ferroptosis hinges on elevated iron levels and lipid peroxide accumulation, countered by antioxidants like GPX4 and GSH. Investigating iron metabolism during viral infection, alongside ferroptosis regulatory mechanisms, can deepen our understanding of viral pathophysiology and provide new therapeutic insights.

#### 4.1.1 Hepatitis viruses

**Figure 2:**
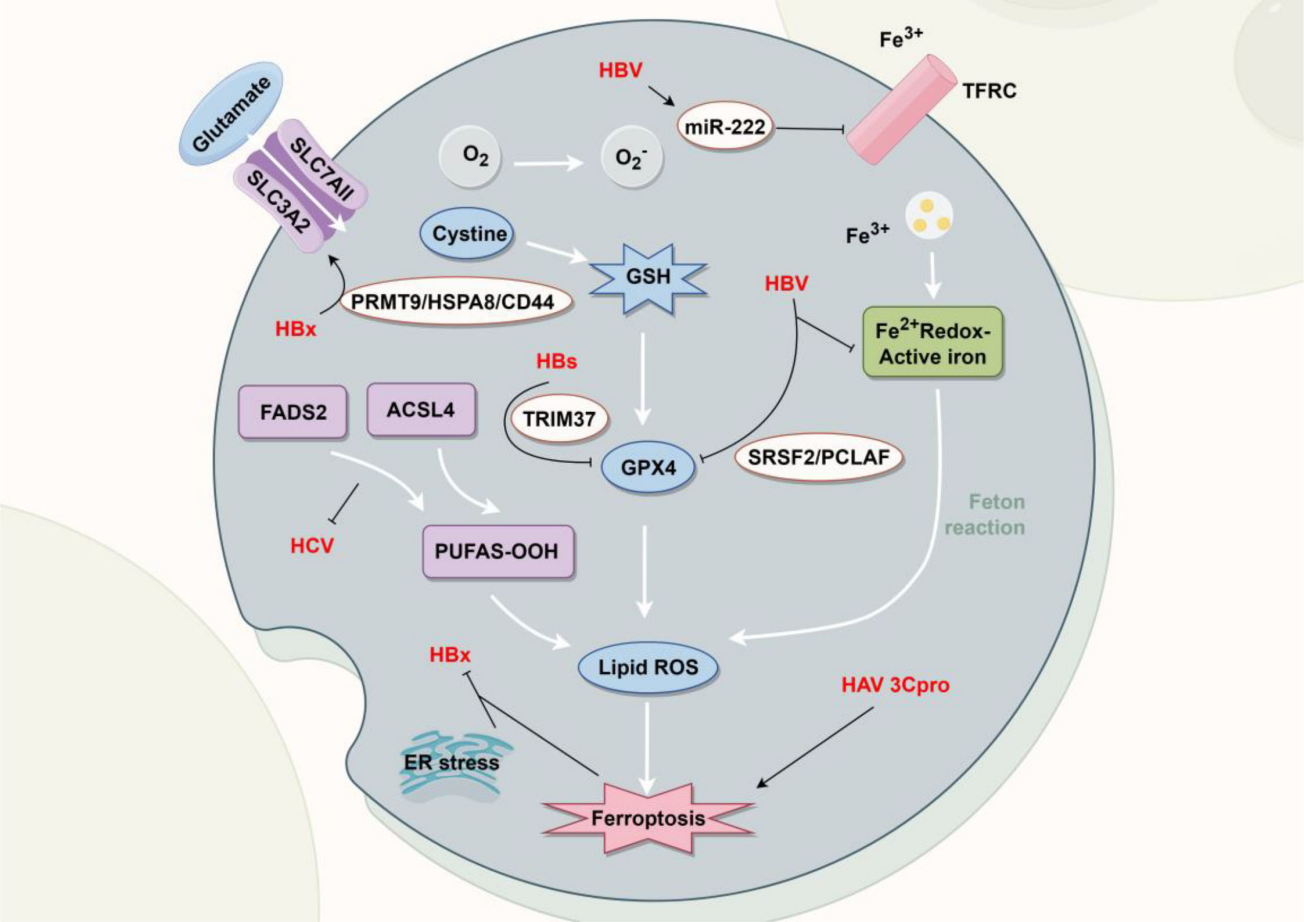
Ferroptosis and hepatitis viruses. Hepatitis B virus (HBV) modulates ferroptosis through multiple mechanisms: HBV can upregulate miR-222, affecting reactive oxygen species (ROS) levels; enhance active iron pool via serine/arginine-rich splicing factor 2 (SRSF2)/proliferating cell nuclear antigen clamp-associated factor (PCLAF); and, via its proteins HBV X protein (HBx) and HBV surface protein (HBs), regulate expression of key mediators such as fatty acid desaturase 2 (FADS2), acyl-CoA synthetase long-chain family member 4 (ACSL4), tripartite motif containing 37 (TRIM37), and factors involved in endoplasmic reticulum (ER) stress. Hepatitis A virus (HAV) 3C protease (3Cpro) promotes ferroptosis by influencing lipid ROS accumulation, while Hepatitis C virus (HCV) disrupts lipid metabolism by targeting enzymes including FADS2. Created by Figdraw. Arrows indicate activation (→) and inhibition (⊣).

Hepatitis viruses, including hepatitis A virus (HAV), HBV, and hepatitis C virus (HCV), cause liver inflammation and damage, leading to acute or chronic hepatitis. Severe cases can result in liver cirrhosis, cancer, or death. These viruses influence ferroptosis in complex ways, impacting cellular and organ function (**Figure 2**). For instance, the HBx protein, a key regulator of viral infection and replication, is also linked to hepatocellular carcinoma (HCC). Deng et al. revealed that HBx induces protein arginine methyltransferase 9 (PRMT9) expression in HCC cells. PRMT9 then targets heat shock protein family A member 8 (HSPA8) and enhances arginine methylation at residues R76 and R100. The resulting elevation in HSPA8 upregulates CD44 expression, collectively suppressing ferroptosis in HBV-associated hepatic cancer cells and thereby promoting tumor progression (Deng et al., 2023). Moreover, hepatic stellate cells (HSCs) are pivotal in the development of liver fibrosis. Upon liver damage or inflammation, activated HSCs transform into myofibroblasts, promoting collagen fiber production and liver connective tissue proliferation. Recent findings indicate that HBV-infected hepatocytes (LO2 cells) secrete extracellular vesicles containing miR-222, which suppresses transferrin receptor (TFRC) expression in HSCs (LX2 cells). This suppression inhibits ferroptosis and promotes stellate cell activation, ultimately leading to liver fibrosis (LF) (Zhang et al., 2023b).

Several studies have affirmed the reciprocal relationship between viral hepatitis infection and ferroptosis. Komissarov et al. (2021) observed that the 3C protein of HAV induces ferroptosis when expressed in isolation in human cells (HEK293, HeLa, and A549) (Komissarov et al., 2021). This form of cell death triggered by the 3C protein can be effectively inhibited by ferroptosis inhibitors, marking the initial evidence that viral proteases can trigger ferroptosis (Komissarov et al., 2021). Pan et al. investigated the connection between HBV and stellate cell ferroptosis. Their findings revealed that hepatitis B surface antigen (HBsAg) promotes N6-methyladenosine modification of tripartite motif containing 37 (TRIM37) mRNA stability, which stabilizes TRIM37 expression. TRIM37 then induces ferroptosis in stellate cells through ubiquitination-dependent mechanisms, reducing cell viability and impairing male fertility (Pan et al., 2023). Additionally, Shi et al. observed that HBV-positive HCC patients with higher serum selenium levels exhibit better prognoses. Through *in vitro* experiments, they determined that low-dose selenium suppresses ferroptosis by upregulating GPX4 expression, thereby attenuating HBV-induced hepatotoxicity (Shi et al., 2023a).

Significant differences exist in the ferroptosis mechanisms of HAV, HBV, and HCV. HBV typically causes chronic infections, suppressing ferroptosis sensitivity in tumor cells, thereby promoting tumor proliferation and liver fibrosis. This capacity for persistence and adaptation is characteristic of HBV-associated cancer cells. In contrast, HAV and HCV primarily induce acute infections and tend to promote viral dissemination by inducing ferroptosis (Komissarov et al., 2021; Yamane et al., 2022). These findings underscore that hepatitis viruses can either hinder or facilitate ferroptosis through diverse mechanisms, offering crucial insights into liver disease progression. Further exploration of this interplay can establish a theoretical foundation for developing novel therapeutic strategies.

#### 4.1.2 Human immunodeficiency virus

**Figure 3:**
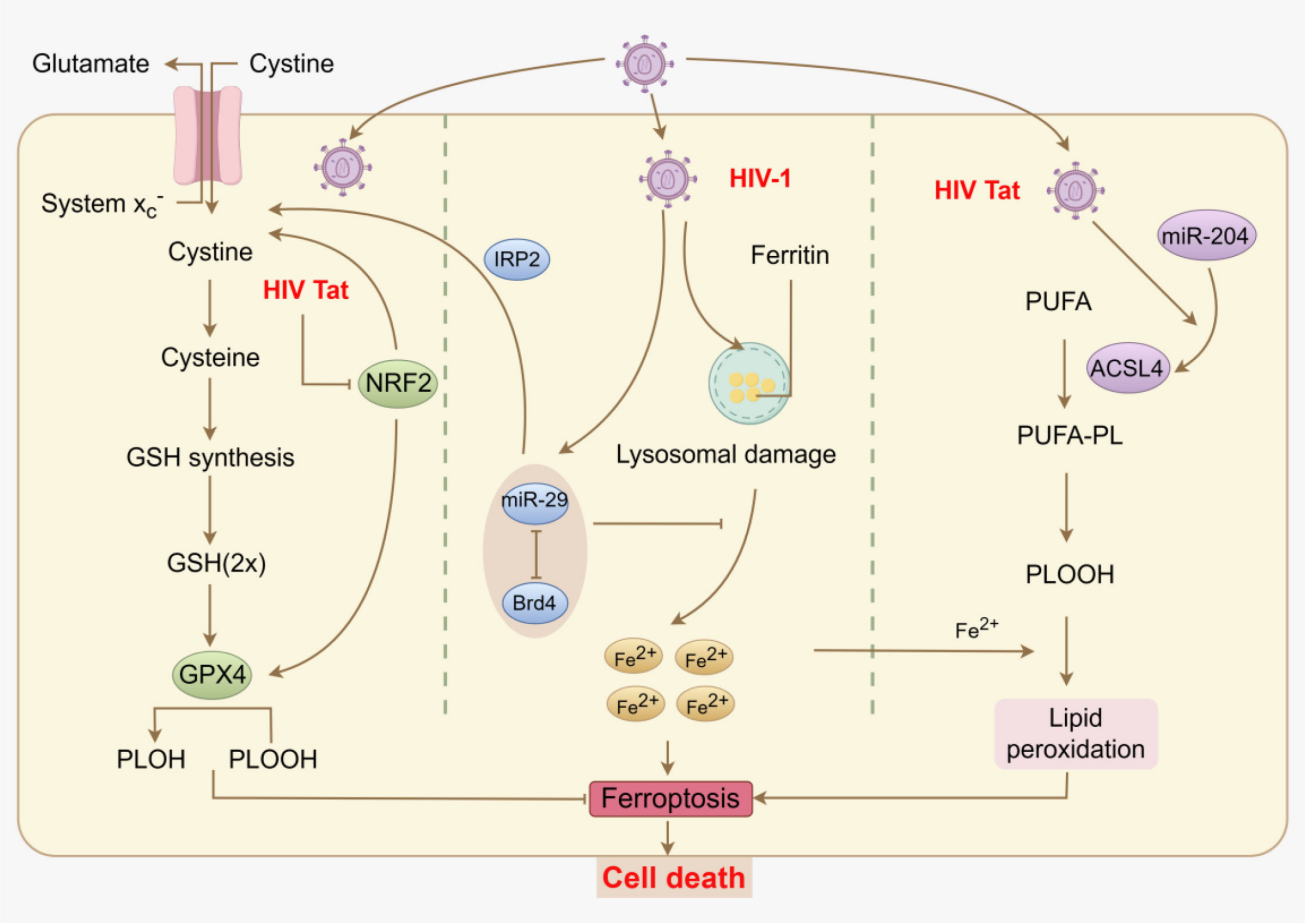
Ferroptosis and human immunodeficiency virus (HIV). The HIV Tat protein inhibits nuclear factor erythroid 2-related factor 2 (NRF2), reducing cysteine availability and glutathione (GSH) synthesis, thereby weakening antioxidant capacity. HIV-1 infection causes ferritin accumulation and lysosomal damage, which releases ferrous iron (Fe^2+^) that promotes lipid peroxidation. miR-29, via bromodomain-containing protein 4 (Brd4) inhibition, further enhances iron-mediated oxidative stress. The Tat protein also upregulates acyl-CoA synthetase long-chain family member 4 (ACSL4) via miR-204, increasing polyunsaturated fatty acid (PUFA) peroxidation, leading to the accumulation of lipid hydroperoxides (PLOOH) and iron overload, and ultimately triggering ferroptosis and cell death. Created by Figdraw. Arrows indicate activation (→) and inhibition (⊣).

Human immunodeficiency virus (HIV) infects the human immune system, resulting in the depletion of crucial immune cells, particularly CD4^+^ T lymphocytes. This gradual immune deterioration increases susceptibility to opportunistic infections, leading to severe complications. The HIV-1 Tat protein, a transcriptional activation protein of HIV, promotes viral gene transcription and replication, while also regulating host cell gene expression, thus impacting host cell biological functions. Kannan et al. discovered that HIV-1 Tat protein upregulates ACSL4 expression via miR-204 (Kannan et al., 2023). This upregulation leads to increased levels of oxidized phosphatidylethanolamine, lipid peroxidation, upregulation of lipase (LIP) and ferritin heavy chain (FTH1), downregulation of GPX4, and mitochondrial outer membrane rupture. Consequently, this process induces ferroptosis in mouse primary microglia (mPMs), a phenomenon also observed in HIV-1 transgenic rats and HIV-positive human brain samples (Kannan et al., 2023). (**Figure 3**).

HIV-associated neurocognitive disorders (HAND) encompass memory and behavioral impairments commonly observed in HIV patients, even during combination antiretroviral therapy. Studies indicate that HIV infection can trigger ferroptosis in neurons and glial cells, contributing to white and gray matter damage and the onset of neurodegenerative pathology (Sfera et al., 2022). Methamphetamine (METH), a potent central nervous system (CNS) stimulant, exacerbates neurotoxicity in HIV patients. Sfera et al. discovered that the combined effect of METH and HIV-1 Tat induces oxidative stress, increasing ferroptosis in BV2 microglial cells, thereby elevating HAND risk. Notably, NFE2 like bZIP transcription factor 2 (NRF2) counters this ferroptosis damage by regulating SLC7A11, offering a potential therapeutic avenue (Sfera et al., 2022). These studies underscore the significance of the HIV-ferroptosis link in understanding the pathophysiological mechanisms underlying HIV-related neurological damage, offering new insights into neurodegeneration mechanisms. Further research in this area may yield novel neuroprotective therapeutic strategies for HIV-related neurological disorders.

#### 4.1.3 Coronaviruses

Coronaviruses are single-stranded positive-sense RNA viruses within the family *Coronaviridae*, infecting both humans and animals. They primarily induce respiratory tract infections, varying from mild symptoms to severe complications like pneumonia. Among the various subtypes, some, such as SARS-CoV, middle east respiratory syndrome coronavirus (MERS-CoV), and the recent SARS-CoV-2, have caused global health crises.

**Figure 4:**
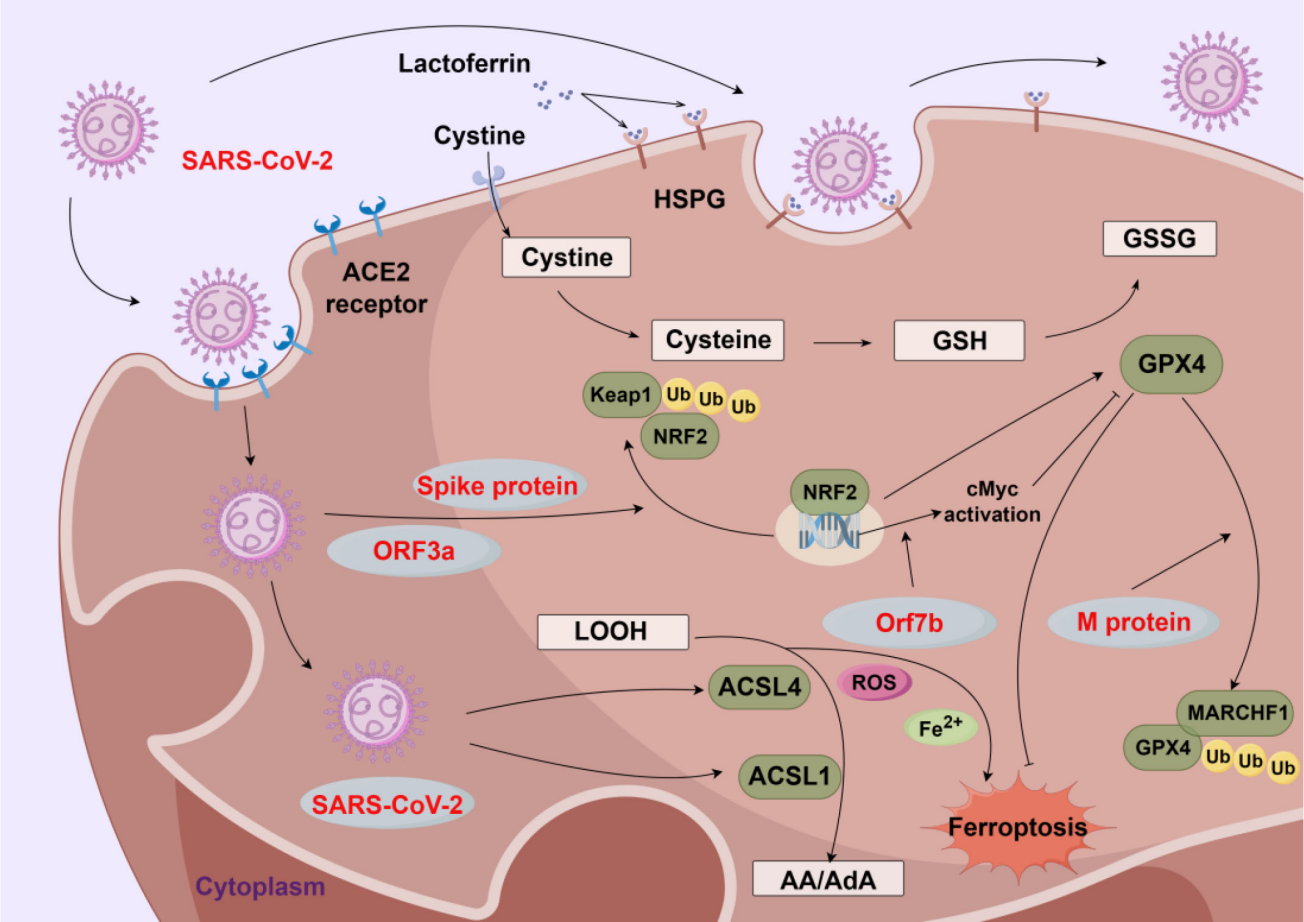
Ferroptosis and coronaviruses. The severe acute respiratory syndrome coronavirus 2 (SARS-CoV-2) enters cells via angiotensin-converting enzyme 2 (ACE2) receptors, with involvement of heparan sulfate proteoglycans (HSPG) and is modulated by extracellular lactoferrin. Viral proteins, including Spike, open reading frame 3a (ORF3a), open reading frame 7b (ORF7b), and M protein, impact cellular redox balance and ferroptosis-related pathways. The Spike protein and ORF3a inhibit nuclear factor erythroid 2-related factor 2 (NRF2)-mediated antioxidant responses, reducing glutathione (GSH) synthesis and weakening glutathione peroxidase 4 (GPX4) activity, while ORF7b activates cellular myelocytomatosis oncogene (cMyc) to disrupt redox homeostasis. M protein promotes GPX4 ubiquitination via membrane associated ring-CH-type finger 1 (MARCHF1), further impairing antioxidant defense. Reduced cysteine and GSH levels enhance lipid peroxidation, with acyl-CoA synthetase long-chain family member 4 (ACSL4) and acyl-CoA synthetase long-chain family member 1 (ACSL1) driving the biosynthesis of peroxidation-prone fatty acids (arachidonic acid/adrenic acid, AA/AdA). Accumulation of lipid hydroperoxides (LOOH) and ferrous iron (Fe^2+^) promotes reactive oxygen species (ROS) generation, ultimately leading to ferroptosis. Created by Figdraw. Arrows indicate activation (→) and inhibition (⊣).

Numerous studies have shown that SARS-CoV-2 infection leads to alterations in lipid metabolism and multi-organ damage (**Figure 4**). Specifically, the spike protein and ORF3a protein of SARS-CoV-2 compromise cellular antioxidant capacity by downregulating NRF2, thereby promoting ferroptosis (Liu et al., 2023b; Nguyen et al., 2022). A study in 2022 pinpointed ACSL4 as a pivotal regulator of ferroptosis, playing a critical role in the formation of replication organelles during SARS-CoV-2 replication. Targeting ACSL4 with drugs like raloxifene and pioglitazone reduces viral load, presenting a novel strategy to inhibit ferroptosis and decrease viral production (Kung et al., 2022). Moreover, Han et al. discovered that SARS-CoV-2 infection induces ferroptosis in human sinoatrial node (SAN)-like pacemaker cells, characterized by ROS accumulation and altered expression of ferroptosis-related genes including SLC7A11, ACSL4, CP, TF, and GPX4. This implicates ferroptosis as a potential mechanism underlying post-SARS-CoV-2 arrhythmias. Additionally, the study identified two candidate drugs, deferoxamine and imatinib, capable of blocking SARS-CoV-2-associated ferroptosis (Nguyen et al., 2022). Recent studies have also shown that SARS-CoV-2 induces ferroptosis through distinct viral proteins: the membrane protein promotes membrane associated RING-CH-Type Finger 1(MARCHF1)/GPX4-mediated ferroptosis by enhancing lipid accumulation, while the accessory protein Orf7b triggers both apoptosis and ferroptosis (Deshpande et al., 2024; Sun et al., 2025). Beyond direct viral protein-mediated ferroptosis, iron overload in SARS-CoV-2 patients directly contributes to hyperferritinemia and systemic inflammation. Consequently, increased ferritin levels might trigger nuclear receptor coactivator 4(NCOA4)-mediated ferritinophagy (Jia et al., 2021; Li et al., 2024a). Furthermore, SARS-CoV-2-induced acute respiratory distress syndrome during pregnancy may result in fetal hypoxia, subsequently triggering tissue acidosis and excessive release of iron from hemoglobin and transferrin, thereby exacerbating lipid peroxidation and ultimately predisposing neonatal neural cells to ferroptosis (Jovandaric et al., 2022). Collectively, these findings highlight the role of the spike protein, ORF3a protein, membrane protein, and accessory ORF7b protein in promoting ferroptosis through multiple pathways, thereby facilitating viral infectivity and replication. Ultimately, the significance of lipid metabolism in regulating ferroptosis during coronavirus infection underscores the need for investigating novel therapeutic interventions.

#### 4.1.4 Influenza viruses

**Figure 5:**
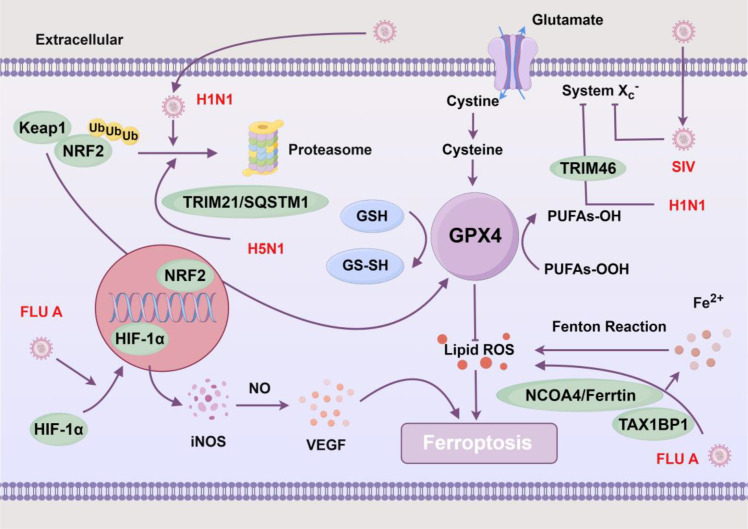
Ferroptosis and influenza viruses. Influenza A virus (FLU A), highly pathogenic avian influenza A virus subtype H5N1(H5N1), pandemic influenza A virus subtype H1N1(H1N1), and swine influenza virus (SIV) can modulate ferroptosis pathways by targeting key molecules. H5N1 and H1N1 promote degradation of nuclear factor erythroid 2-related factor 2 (NRF2), impairing cellular redox defense. FLU A activate hypoxia-inducible factor 1-alpha (HIF-1α), resulting in increased inducible nitric oxide synthase (iNOS) and vascular endothelial growth factor (VEGF) expression, further contributing to oxidative and nitrosative stress. Viral such as SIV and H1N1 can inhibit cystine import through system x_c_^⁻^, limiting cysteine and glutathione (GSH) synthesis and weakening glutathione peroxidase 4 (GPX4)-mediated antioxidant protection. Tax1 binding protein 1 (TAX1BP1)/nuclear receptor coactivator 4 (NCOA4)-mediated ferritinophagy increases free Fe^⁺^, fueling the Fenton reaction and generating lipid reactive oxygen species (ROS). These processes culminate in lipid peroxidation, ROS accumulation, and ferroptosis during influenza virus infection. Created by Figdraw. Arrows indicate activation (→) and inhibition (⊣).

Influenza viruses, including influenza A virus (FLU A) and influenza B virus (FLU B), are single-stranded negative-sense RNA viruses classified under the Orthomyxoviridae family. These viruses predominantly spread via respiratory droplets, affecting the upper respiratory tract and potentially leading to severe complications like pneumonia and mortality. FLU A notably activates HIF-1, influencing ferroptosis-related metabolism and the expression of key proteins like ACSL4 and GPX4. This activation induces ferroptosis in mouse lung epithelial (MLE-12) cells, contributing to lung congestion, edema, and inflammation (Huang et al., 2023). (**Figure 5**) Moreover, H1N1 infection triggers differential expression of ferroptosis-related genes and metabolites in human nasal epithelial progenitor cells (hNEPCs). Through upregulation of NRF2/Kelch-like ECH-associated protein 1 (KEAP1) expression, H1N1 modulates glutamine metabolism in hNEPCs, inducing ferroptosis and nasal mucosal epithelial inflammation (Liu et al., 2023a). Beyond its effects on nasal epithelial cells, H1N1 infection also accelerates ferroptosis and lung injury via tripartite motif containing 46 (TRIM46)-mediated ubiquitination of SLC7A11 (Zhou et al., 2024a). In mouse models, the glutamine inhibitor JHU-083 effectively mitigates H1N1-induced immune system damage, presenting a promising therapeutic strategy for virus-induced nasal inflammation (Liu et al., 2023a). Ouyang et al. demonstrated that FLU A hemagglutinin interacts with NCOA4 and Tax1 binding protein 1 (TAX1BP1) to promote ferritinophagy and the formation of ferritin-NCOA4 condensates, thereby facilitating viral replication (Ouyang et al., 2024). Additionally, another study from Wei et al. showed that H5N1 triggers oxidative stress and ferroptosis through TRIM21-mediated regulation of the sequestosome 1 (SQSTM1/p62)-NRF2-KEAP1 axis, further facilitating viral replication (Wei et al., 2024). Notably, ferroptosis-related disruptions are not confined to human-infecting strains alone. Swine influenza virus (SIV), an influenza A virus prevalent in swine populations, can also infect humans under certain conditions, causing zoonotic infections. Research indicates that SIV infection disrupts intracellular iron metabolism and suppresses SLC7A11/GPX4 axis activation in A549 cells. Consequently, this disruption promotes cellular lipid peroxidation and iron-dependent cell death, facilitating viral replication (Cheng et al., 2022). Collectively, these findings underscore the intricate relationship between influenza virus infection and ferroptosis mechanisms, particularly in disrupting epithelial cells of the upper respiratory tract and lungs, thereby enhancing viral replication. Understanding this interaction is crucial for unraveling the pathophysiology of viral infections and may reveal novel therapeutic strategies for influenza.

#### 4.1.5 Herpesviruses

**Figure 6:**
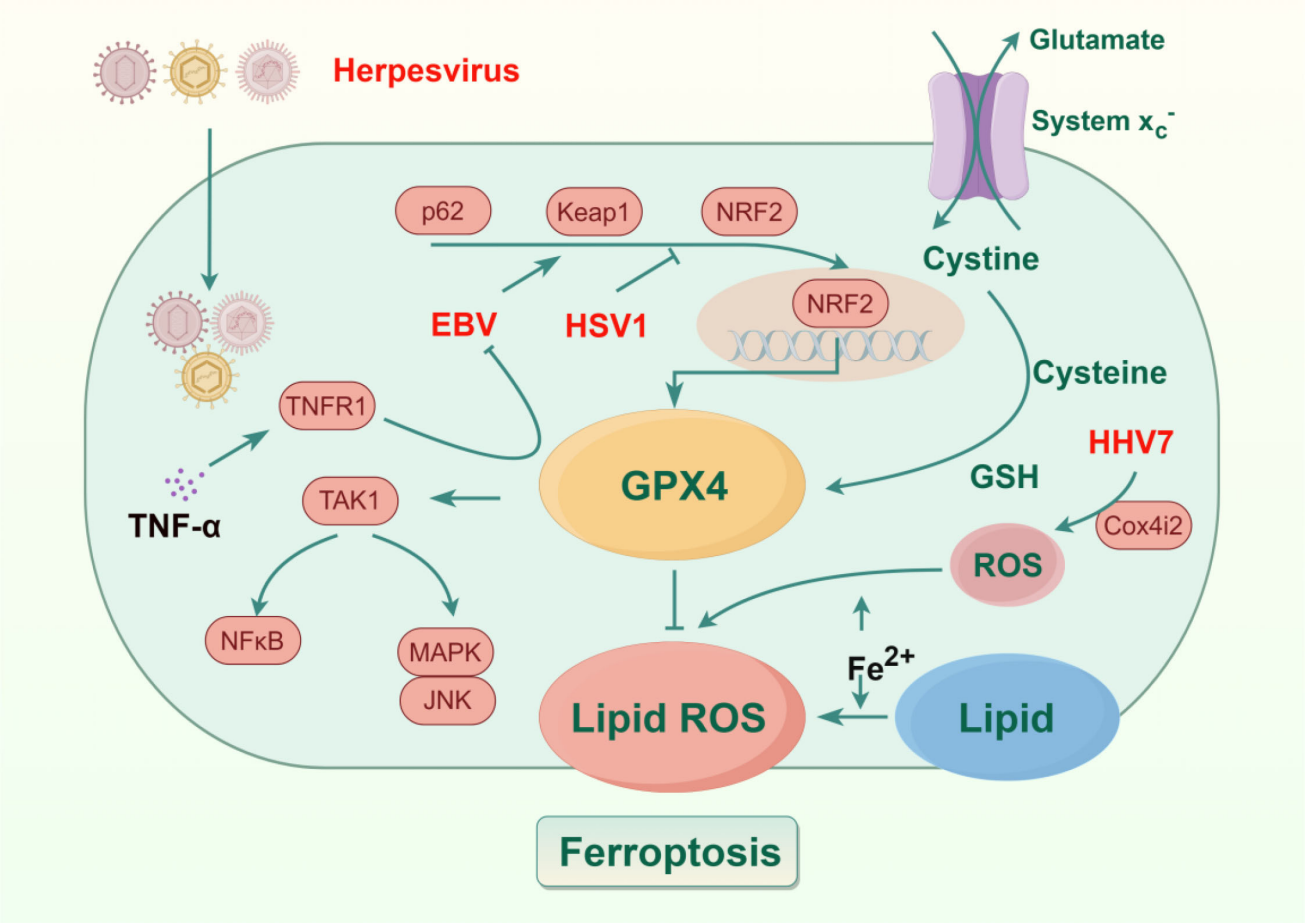
Ferroptosis and herpesviruses. Epstein-Barr virus (EBV) and herpes simplex virus 1 (HSV1) interfere with glutathione peroxidase 4 (GPX4) activity directly or through modulation of sequestosome 1 (SQSTM1/p62) - Kelch-like ECH-associated protein 1 (Keap1)- nuclear factor erythroid 2-related factor 2 (NRF2), decreasing the cellular capacity to clear lipid peroxides, subsequently triggering pathways such as nuclear factor kappa-light-chain-enhancer of activated B cells (NF-κB) and mitogen-activated protein kinase(MAPK)/c-Jun N-terminal kinase (JNK) through transforming growth factor-β-activated kinase 1 (TAK1). Human herpesvirus 7 (HHV-7) enhances oxidative stress via cytochrome c oxidase subunit 4 isoform 2 (Cox4i2), promoting ROS accumulation. Excess iron (Fe^2+^) and the resulting lipid ROS drive lipid peroxidation, ultimately leading to ferroptosis during herpesvirus infection. tumor necrosis factor-alpha (TNF-α) recognizes tumor necrosis factor receptor 1 (TNFR1) and inhibits EBV infection by upregulating the expression of GPX4. Created by Figdraw. Arrows indicate activation (→) and inhibition (⊣).

Herpesviruses, including herpes simplex virus types 1 and 2 (HSV-1/2), varicella zoster virus (VZV), EBV, and human herpesviruses 6, 7, and 8 (HHV-6/7/8), are DNA viruses within the Herpesviridae family. These viruses are responsible for a range of human diseases, and recent studies suggest that ferroptosis may play a crucial role in their pathogenesis (**Figure 6**). Yuan et al. discovered that EBV activates the p62/KEAP1/NRF2 signaling pathway, which in turn increases the expression of SLC7A11 and GPX4. This upregulation reduces the sensitivity of nasopharyngeal carcinoma (NPC) cells to ferroptosis, thereby fostering chemoresistance and tumor progression (Yuan et al., 2022). Building on this, Zhang et al. found that TNF-α can inhibit EBV reactivation by acting on tumor necrosis factor receptor 1 (TNFR1) and modulating the GPX4-mediated ferroptosis pathway (Zhang et al., 2025). In contrast to EBV’s tumor-associated mechanisms, HSV-1 can cause encephalitis, leading to brain inflammation and neurological dysfunction. Typically, this occurs when the virus reactivates during oral herpes recurrences and enters the central nervous system. A 2023 study revealed that HSV-1 infection enhances the ubiquitination and degradation of NRF2 by the E3 ubiquitin ligase Keap1, disrupting cellular redox homeostasis and promoting ferroptosis in the mouse central nervous system. This ferroptosis leads to CNS inflammation. Notably, ferroptosis inhibitors or proteasome inhibitors can effectively alleviate HSV-1-associated encephalitis by inhibiting NRF2 degradation (Xu et al., 2023). Extending the neurological implications of herpesvirus-induced ferroptosis, HHV-7 infections are mild and asymptomatic. However, Chang et al. highlighted that HHV-7 infection of Schwann cells in the peripheral nervous system induces oxidative stress through cytochrome c oxidase subunit 4I2 (Cox4i2) and regulates ferroptosis-related gene expression via the extracellular regulated mitogen-activated protein kinase (MAPK) signaling pathway. This induction of ferroptosis in Schwann cells promotes neuroinflammation, leading to facial nerve damage (Chang et al., 2021). Taken together, further exploration of the herpesvirus-ferroptosis relationship may elucidate cellular biological processes underlying disease development, offering insights for more effective treatment strategies.

Overall, as a carcinogenic virus, EBV shares similar mechanisms with HBV, both of which facilitate tumor progression and induce chemotherapy resistance by suppressing ferroptosis sensitivity in tumor cells. In contrast, herpesviruses including HSV-1 and HHV-7 more closely resemble coronaviruses and influenza viruses in promoting viral infection through ferroptosis induction. This process is closely related to the inflammatory responses observed in both acute and chronic infections. In the acute phase, the inflammatory response can enhance iron metabolism, thereby facilitating ferroptosis. Conversely, in chronic infections, persistent inflammation may dysregulate ferroptosis as a consequence of viral immune evasion, thereby creating a favorable environment for the continuous proliferation and spread of the virus.

#### 4.1.6 Other viruses

Ferroptosis is implicated in various viral infection processes, including tumor cell death triggered by Newcastle disease virus (NDV), encephalitis caused by JEV, and tissue damage induced by human adenovirus type 7 (HAdV-7), enterovirus A71(EV-A71) and et al (Chooi et al., 2024; Kan et al., 2021; Kung et al., 2022; Yang et al., 2022; Zhao et al., 2024b; Zhu et al., 2023, Zhu et al., 2024). Notably, NDV, classified as an oncolytic virus, exploits ferroptosis as an antitumor mechanism by promoting E3 ubiquitin ligase parkin (PRKN)-mediated ubiquitination and degradation of Yes-associated protein (YAP) at Lysine 90 (Lys90) while concurrently activating the p53/SLC7A11/GPX4 pathway, thereby intensifying ferroptosis in tumor cells (Kan et al., 2021; Sun et al., 2024). This highlights the critical role of oncolytic viruses in cancer therapy. Similarly, both JEV and HAdV-7 infection have been demonstrated to induce ferroptosis through upregulation of iron-dependent lipid peroxidation and suppression of antioxidant defenses, most notably GPX4 downregulation (Yang et al., 2022; Zhou et al., 2024b; Zhu et al., 2024). Collectively, both DNA and RNA viruses can promote viral replication and induce inflammatory responses by triggering ferroptosis. Together, these findings illustrate the central involvement of ferroptosis in diverse aspects of viral pathogenesis, highlighting its potential as a pivotal mechanism underlying virus-induced cellular damage.

The interplay between virus and ferroptosis is a burgeoning area of research that promises to unveil novel insights into the cellular processes underlying disease development. Understanding how these viruses manipulate ferroptosis can not only deepen our comprehension of their pathogenic mechanisms but also pave the way for innovative treatment strategies. As research progresses, these findings will provide a fresh perspective on viral pathogenesis and drug development, potentially leading to more effective therapeutic interventions for a range of viral infections (Chen et al., 2023b; Li et al., 2023c; Zhang et al., 2023a). **Table 1** summarizes the diverse roles of ferroptosis in viral infections, revealing how different viruses either promote or suppress ferroptosis to facilitate infection or tumor progression.

**Table 1:** List of the mechanisms between the viruses and ferroptosis.

Pathogens	Mechanisms	Experimental models	Outcomes	Reference
HAV	Induce ferroptosis by 3Cpro	293T, Hela	Enhance virus replication	(Komissarov et al., 2021)
HBV	Regulate GPX4	HSCs	Alleviate hepatic stellate cell fibrosis	(Kuo et al., 2020)
	Mediate GPX4 ubiquitination by TRIM37	Human Sertoli cells	Reduce the viability of human support cells	(Pan et al., 2023)
	Regulate the SRSF2/PCLAF tv1 axis	HCC	Induce sorafenib resistance	(Liu et al., 2023c)
	Regulate the miR-222/TFRC axis	LO2, LX-2	Promote liver fibrosis	(Zhang et al., 2023b)
	Regulate HBx/PRMT9/HSPA8/CD44 axis	HCC	Promote cancer progression	(Deng et al., 2023)
	Upregulate SLC7A11/GPX4	HCC	Promote cancer progression	(Wang et al., 2023c)
HCV	Regulate ferroptosis through FADS2	Huh7.5, 293FT, PH5CH8, A549	Inhibit virus replication	(Yamane et al., 2022)
HIV	Regulate ferroptosis-driving molecules	/	Facilitate the occurrence of neurodegenerative diseases	(Sfera et al., 2022)
	HIV-1 Tat combined with METH	BV2	Enhance virus replication	(Lin et al., 2023)
	Regulate miR-204/ACSL4 pathway	mPMs	Enhance virus replication	(Kannan et al., 2023)
SARS-CoV-2	Iron overload	/	Enhance virus replication	(Habib et al., 2021)
	Via ORF3a/Keap1/NRF2 axis	NCI-1299, A549-ACE2	Enhance virus replication	(Liu et al., 2023b)
	Regulate MARCHF1/GPX4 axis	Hela	Enhances viral replication	(Sun et al., 2025)
	Orf7b Induces ferroptosis	Beas-2B	Induces lung injury	(Deshpande et al., 2024)
M-CoV	Promote ferroptosis by ACSL1	RAW 264.7, iBMDM	Enhance virus replication	(Xia et al., 2021)
H1N1	Regulate NRF2/KEAP1/GCLC axis	hNEPCs	Promote inflammation	(Liu et al., 2023a)
	TRIM46 regulate SLC7A11 ubiquitination	A549, 16HBE	Induces lung injury	(Zhou et al., 2024a)
FLU A	FLU A-HA interacts with NCOA4 and TAX1BP1	A549	Enhance virus replication	(Ouyang et al., 2024)
H5N1	TRIM21 Promotes ferroptosis through the SQSTM1/NRF2/KEAP1 Axis	A549	Increase the titers of virus	(Wei et al., 2024)
SIV	Inhibit the Xc-/GPX4 axis	A549	Enhance virus replication	(Cheng et al., 2022)
HSV-1	Regulate the NRF2/Keap1 pathway	U373, HMC3	Promote viral encephalitis	(Xu et al., 2023)
HHV-7	Induce oxidative stress by Cox4i2	SCs	Enhance virus replication	(Chang et al., 2021)
EBV	Disrupt the antioxidant defense mechanism	Primary B Cell	Promote lymphoma progression	(Burton et al., 2022)
	TNF-α affect the GPX4 protein	Raji and LCL	Inhibits viral reactivation	(Zhang et al., 2025)
	Activate the p62/Keap1/NRF2 pathway	NPC	Reduce tumor cell sensitivity to chemotherapy drugs	(Yuan et al., 2022)
ZIKV	Regulate YBB, HMOX1, SALT, and SLAC40A1	C6/36	Not clear	(Feng et al., 2023)
	Regulate proteasome complex and an E3 ligase NEDD4	Mice	Reduce virus infection	(Zhao et al., 2024a)
CVB3	Recruit nuclear Sp1 to upregulate the expression of TFRC	Hela	Enhance virus replication	(Yi et al., 2022)
	Ferroptosis occurs through crosstalk with complement C4 through interaction with TFRC	Mice	Promote inflammatory responses	(Jiawang et al., 2024)
CVA6	ACSL4 is involved in viral replication complex formation	RD, 293T, A549	Enhance virus replication	(Kung et al., 2022)
EV-A71	Target Nrf2/SLC7A11/GSH pathway	RD, Hela	Inhibit virus infection	(Zhao et al., 2024b)
	Induce neurodegeneration by ferroptosis	H7 embryonic stem cells	Neurodegeneration	(Chooi et al., 2024)
LCMV	Induce virus-specific memory CD4+ T cells to undergo ferroptosis	CD^4+^ T cells	Enhance virus replication	(Wang et al., 2022)
RV	Regulate the SLC7A11-AS1/xCT axis	HT-29	Enhance virus replication	(Banerjee et al., 2024)
DENV	Regulate JUN, IL6, ATF3, XBP1, and CDKN2A	HepG2	Not clear	(Li et al., 2023b)
PEDV	RSL3 Inhibit viral replication	Vero	Inhibit virus infection	(Li et al., 2023c)
ASFV	Reduce glutathione levels	PAMs	Enhances viral replication	(Gao et al., 2024a)
	Brequinar inhibit viral replicatio	PAMs	Inhibit virus infection	(Chen et al., 2023b)
NDV	Regulate the p53/SLC7A11/GPX4 axis	U251	Promote tumor cell death	(Kan et al., 2021)
	By inducing ubiquitin-mediated degradation of YAP at Lys90 through E3 ubiquitin ligase PRKN	Cancer cells	Promote tumor cell death	(Sun et al., 2024)
JEV	Inhibit GSH/GPX4 and promote YAP1/ACSL4 axis	BHK-21, Hela, BV2, SY5Y	Enhance virus replication	(Zhu et al., 2024)
	Ferroptosis agonist can suppress JEV proliferation, whereas the ferroptosis antagonist promotes JEV proliferation	PK-15	Enhance virus replication	(Zhou et al., 2024b)
	Inhibit GPX4/GSH, as well as promote lipid peroxidation mediated by YAP1/ACSL4	BV2, SH-SY5Y	Induced neuronal damage and neuroinflammation	(Zhu et al., 2023)
BVDV	Via Nrf2-GPX4 pathway and NCOA4-mediated ferritinophagy	Madin-Darby bovine kidney cells	Promote inflammatory responses	(Li et al., 2024b)
FAdV-4	Via the p53-SLC7A11-GPX4 axis	LMH cell	Induce fatty liver	(Dong et al., 2024)
HERVs	Downregulate the expression of GPX4 and SLC3A2	SY5Y	Contribute to neuronal cell death	(Zhang et al., 2024a)

HAV, Hepatitis A Virus; HBV, Hepatitis B Virus; HCV, Hepatitis C Virus; HIV, Human Immunodeficiency Virus; SARS-CoV-2, Severe Acute Respiratory Syndrome Coronavirus 2; M-CoV, Murine Coronavirus; H1N1, pandemic influenza A virus subtype H1N1; FLU A, influenza A virus; H5N1, highly pathogenic avian influenza A virus subtype H5N1; SIV, Swine Influenza Virus; HSV-1, Herpes Simplex Virus Type 1; HHV-7, Human Herpesvirus 7; EBV, Epstein-Barr Virus; ZIKV Zika Virus; CVB3, Coxsackievirus B3; CVA6, Coxsackievirus A6; EV-A71, Enterovirus A71; LCMV, Lymphocytic Choriomeningitis Virus; RV, Rotavirus; DENV, Dengue Virus; PEDV, Porcine Epidemic Diarrhea Virus; ASFV, African Swine Fever Virus; NDV, Newcastle Disease Virus; JEV, Japanese encephalitis virus; BVDV, Bovine viral diarrhea virus; FAdV-4, Fowl adenovirus serotype 4; HERVs, Human endogenous retroviruses.

In summary, as detailed in **Table 1**, viral pathogens exhibit divergent strategies in manipulating ferroptosis based on their pathogenic characteristics. Oncogenic viruses (HBV and EBV) facilitate tumor proliferation by suppressing ferroptosis in cancer cells or reducing their sensitivity to anti-tumor drugs, a process closely related to the persistent infection of host cells, which induces chronic cellular stress and inflammation that can drive malignant transformation. In contrast, NDV, as an oncolytic virus, induces ferroptosis to promote tumor cell death, suggesting that NDV may have potential therapeutic applications in cancer treatment. Notably, non-oncogenic viruses, including both DNA and RNA viruses such as SARS-CoV-2, murine coronavirus (M-CoV), HSV-1, HHV-7, H1N1, and coxsackievirus B3 (CVB3), primarily enhance their replication within host cells by inducing ferroptosis, facilitating effective host infection. Notably, changes in ferroptosis-related biomarkers were observed in the clinical samples from patients infected with dengue virus (DENV) and SARS-CoV-2, as identified through bioanalytical data (Li et al., 2023b; Zhao et al., 2023c). However, there are no relevant clinical studies on the effects of ferroptosis in other viruses. Additionally, ferroptosis inhibitors, such as ferrostatin-1 (Fer-1), deferoxamine (DFO), and JHU-083 (a glutaminase antagonist), can suppress the replication of these viruses, demonstrating their antiviral properties. Moreover, neurodegenerative diseases associated with HIV-1 and EV-A71 are closely linked to the occurrence of ferroptosis. Compound therapies, such as RSL3 (a GPX4 inhibitor), brequinar (BQR, brequinar (BQR, a DHODH inhibitor), and metastable iron sulfides (mFeS), can also induce ferroptosis, thereby inhibiting infections of other viruses (porcine epidemic diarrhea virus (PEDV), African swine fever virus (ASFV), and H1N1). This inhibition is likely achieved by activating host cell death program, which eliminates infected cells and reduces viral survival and transmission. Overall, this mechanism highlights the dual role of ferroptosis in the infection process and its potential as a therapeutic strategy to suppress viral replication.

### 4.2 Ferroptosis in bacterial infections

**Figure 7:**
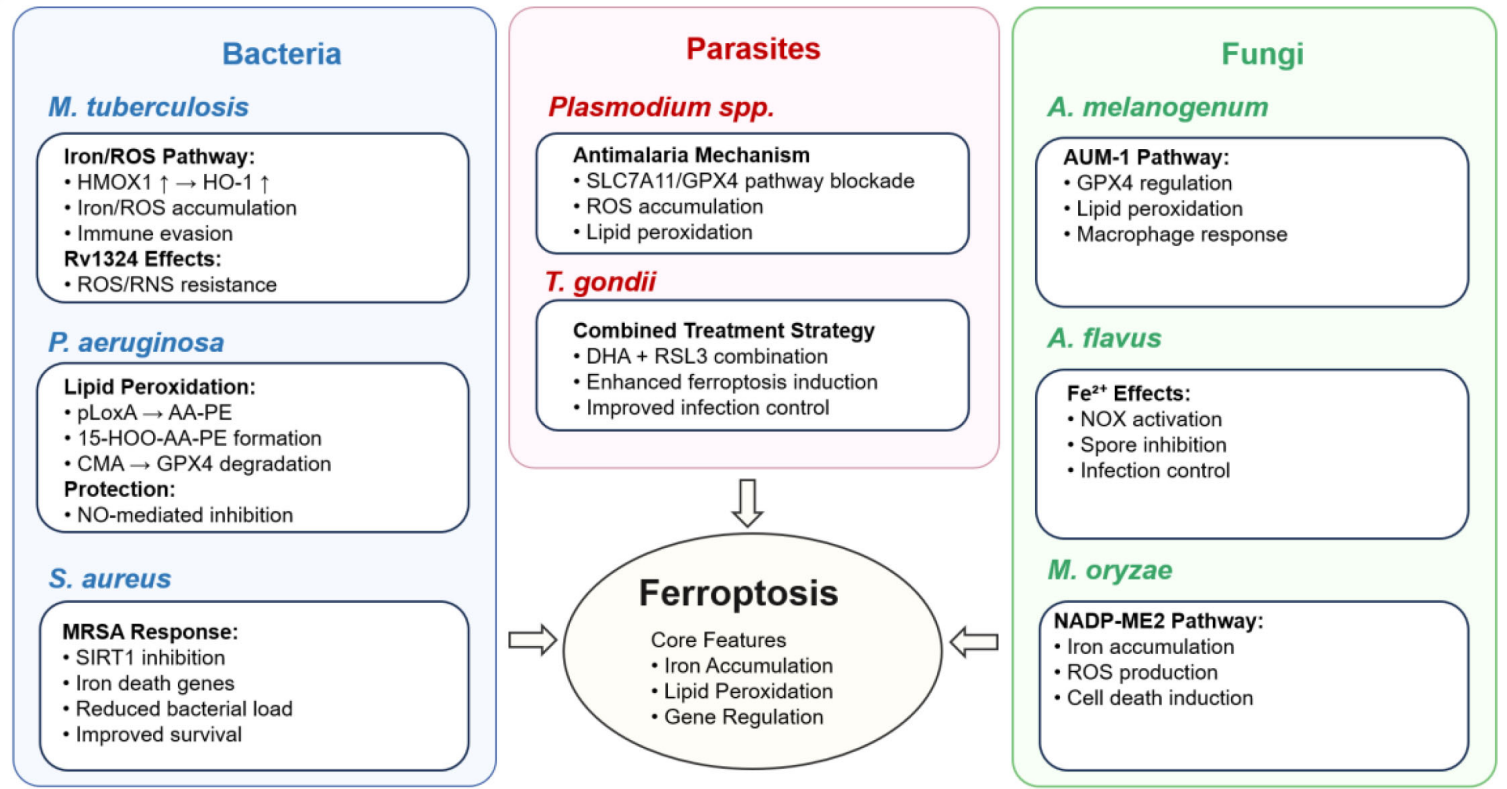
The role of ferroptosis in bacterial, parasitic, and fungal infections is illustrated in this figure, highlighting how different pathogens manipulate ferroptosis pathways to either evade immune responses or contribute to disease control. ROS (reactive oxygen species), HMOX1 (heme oxygenase 1 gene), HO-1 (heme oxygenase 1 protein), pLoxA (*Pseudomonas aeruginosa* lipoxygenase A), AA-PE (arachidonic acid-phosphatidylethanolamine), 15-HOO-AA-PE (15-hydroperoxy-arachidonic acid-phosphatidylethanolamine), CMA (chaperone-mediated autophagy), GPX4 (glutathione peroxidase 4), NO (nitric oxide), MRSA (methicillin-resistant *Staphylococcus aureus*), SIRT1 (sirtuin 1), SLC7A11 (solute carrier family 7 member 11), DHA (dihydroartemisinin), RSL3, AUM-1 (autophagy-related ubiquitin modifier 1), Fe^2+^ (ferrous iron), NOX (NADPH oxidase), NADP-ME2 (NADP-dependent malic enzyme 2). Arrows indicate activation (→) and upregulation (↑).

The pathogenesis of bacterial infections hinges on both host cell status and bacterial survival strategies, metabolic activities, and host interactions, enabling bacteria to thrive in both intracellular and extracellular environments. Maintaining host cell integrity is crucial for preventing bacterial dissemination and facilitating immune cell migration to infection sites. In this context, ferroptosis, a form of regulated cell death characterized by lipid peroxidation and membrane damage, has been implicated in host tissue damage during bacterial infections (**Figure 7**). Indeed, understanding the interplay between bacterial infections and ferroptosis is vital for developing innovative therapeutic strategies and comprehending infectious disease pathogenesis.

#### 4.2.1 Mycobacterium tuberculosis

*M. tuberculosis*, an acid-fast bacillus, resides intracellularly, has a unique lipid-rich cell wall that grants resilience against antibiotics and facilitates evasion of the human immune system. As the causative agent of tuberculosis, *M. tuberculosis* interacts primarily with macrophages, which are critical for infection clearance and inflammation regulation. This interaction is pivotal for tuberculosis pathogenesis and treatment. *M. tuberculosis* invades macrophages, forming mycobacterial granulomas that restrict bacillus growth and dissemination. However, these structures also foster *M. tuberculosis*’s long-term survival within the host.

Recent studies have shown that *M. tuberculosis* evades immune attacks by inducing ferroptosis in macrophages. For instance, Ma et al. conducted transcriptomic analyses of peripheral blood from tuberculosis patients, revealing upregulation of heme oxygenase-1 (HO-1) and pro-ferroptosis factors, alongside downregulation of key anti-lipid peroxidation factors like GPX4 (Ma et al., 2022a). These clinical data further suggest that ferroptosis may play a significant role in M. tuberculosis infection. This hypothesis was subsequently validated in experiments using RAW264.7 mouse macrophage-like cells and in mice infected with Bacillus Calmette-Guérin (BCG). *M. tuberculosis* infection induced ferroptosis through HO-1-mediated regulation of ROS production and iron metabolism, enabling immune escape (Ma et al., 2022a). Beyond iron metabolism dysregulation, the Rv1324 protein secreted by *M. tuberculosis*, a sulfur-redox protein with oxidoreductase activity, disrupts the host cell’s immune response and enhances the survival and replication of *M. tuberculosis* (Devasundaram et al., 2014; Wolfe et al., 2010). Building on this, Shi et al. discovered that the Rv1324 protein not only sustains the presence of *M. tuberculosis* in mouse lung tissue but also enhances its resistance to ROS and reactive nitrogen species (RNS), thereby promoting ferroptosis in lung tissue, leading to pathological damage and inflammation in the mouse lung (Shi et al., 2023b). Taken together, *M. tuberculosis* evades host immune attacks by inducing ferroptosis in macrophages. At the same time, the specific proteins secreted by the bacteria further promote ferroptosis, leading to lung tissue damage and disruption of the host immune response. This dual mechanism not only enhances the survival capacity of the bacteria but also reveals the complex interactions between the host and the pathogen. These insights advance our understanding of the pathogenic mechanisms of *M. tuberculosis* and offer potential new targets for tuberculosis treatment.

#### 4.2.2 Pseudomonas aeruginosa

*P. aeruginosa* is a highly resistant and pathogenic bacterium, often responsible for nosocomial lung infections. It attaches to host cell surfaces using surface adhesins and forms biofilms, which are difficult to eliminate. *P. aeruginosa* produces exotoxins and proteases that damage host cells, suppress macrophage function, and interfere with host immune responses. Emerging evidence suggests that ferroptosis represents a key mechanism through which *P. aeruginosa* exerts its pathogenic effects. Dar et al. discovered that a *P. aeruginosa* strain lacking arachidonic acid-phosphatidylethanolamines (AA-PE) can convert AA-PE in host cells to 15-hydroperoxy-arachidonic acid-phosphatidylethanolamines (15-HOO-AA-PE) via its lipoxygenase (pLoxA), leading to lipid peroxidation and ferroptosis in human bronchial epithelial (HBE) cells during infection (Dar et al., 2022). Consistently, clinical *P. aeruginosa* isolates induce ferroptosis in HBE cells, with GSH levels negatively associated with pLoxA levels and enzyme activity (Dar et al., 2022). Additionally, *P. aeruginosa* activates chaperone-mediated autophagy (CMA), degrading host GPX4 defense and promoting lipid peroxidation-induced ferroptosis in epithelial cells. Notably, the host is not entirely defenseless against these ferroptosis insults; macrophage-produced nitric oxide (NO) defends against ferroptosis by inhibiting 15-HpETE-PE signals, thereby preventing *P. aeruginosa*-induced epithelial cell ferroptosis (Dar et al., 2021). Collectively, these findings highlight that *P. aeruginosa* exploits lipid metabolism dysregulation as a central strategy to induce ferroptosis in host epithelial cells, while host-derived NO partially counterbalances this process, together offering promising therapeutic targets for *P. aeruginosa*-associated pulmonary diseases.

#### 4.2.3 Staphylococcus aureus

*S. aureus*, a Gram-positive intracellular pathogen, causes a variety of infections, including skin, respiratory tract, and bloodstream infections. Methicillin-resistant strains *S. aureus* (MRSA) exhibits resistance to beta-lactam antibiotics. In pulmonary infections, MRSA strains invade lung tissues, causing severe illness. Notably, research using a murine MRSA pneumonia model suggests that inducing MRSA death via ferroptosis characteristics can enhance mouse survival rates, reduce bacterial loads, and mitigate inflammatory damage, offering promising clinical therapeutic implications (Hu et al., 2023a). Beyond pulmonary infections, *S. aureus* commonly causes infectious mastitis. Studies indicate that inhibiting sirtuin 1 (SIRT1) can regulate ferroptosis-related gene expression, curbing inflammation and suppressing *S. aureus*-induced mastitis (Zhao et al., 2023a; Zhao et al., 2023b).

The ferroptosis-inducing capacity of bacterial pathogens extends well beyond *M. tuberculosis*, *P. aeruginosa* and *S. aureus.* Various bacteria, including *Escherichia coli* (*E. coli*), *Salmonella pullorum* (*S. pullorum*), *Listeria monocytogenes* (*L. monocytogenes*), and *Helicobacter pylori* (*H. pylori*), induce iron accumulation and lipid peroxidation during host cell infection, leading to ferroptosis. This process facilitates immune evasion and inflammatory responses by the pathogens and may also induce tumor cell death, offering potential for immunotherapy (Chen et al., 2023a; Hu et al., 2023b; Kwun and Lee, 2023; Ma et al., 2022b; Wang et al., 2023b; Wen et al., 2022; Yan et al., 2023; Zhou et al., 2022). Collectively, ferroptosis plays a significant role in the pathogenesis of bacterial infections by contributing to host tissue damage and influencing immune responses. Understanding the mechanisms by which bacteria induce ferroptosis can provide new therapeutic targets and strategies for combating infectious diseases. **Table 2** outlines the bacteria covered and explains how ferroptosis influences the progression of infections.

**Table 2:** List of the mechanisms between the bacteria and ferroptosis.

Pathogens	Mechanisms	Experimental models	Outcomes	Reference
*M. tuberculosis*	Inhibit GPX4 and promote LPO	Macrophage, mice	Promote lung injury in mice	(Amaral et al., 2019)
	Regulate ferroptosis by HO-1	RAW264.7, mice	Promote bacterial infection	(Ma et al., 2022a)
	Influence ROS and RNS	Mice	Induce pathological damage and inflammation in the lungs	(Shi et al., 2023b)
	Mediated by CYBB	Macrophage	Promote macrophage M2 activation	(Wang et al., 2023d)
	Bach1 can promote lipid peroxidation by decreasing glutathione levels and Gpx4 expression	Mice	Promotes tissue necrosis	(Amaral et al., 2024)
*P. aeruginosa*	Regulate lipid metabolism via pLoxA	HBE	Promote bacterial infection	(Dar et al., 2018)
	Increase ROS and induce LPO	Human cystic fibrosis bronchial epithelial cells	Promote cell death	(Ousingsawat et al., 2021)
	Activate CMA to degrade GPX4 defense mechanism	Macrophage	Promote inflammation	(Dar et al., 2021)
	Inhibit GPX4/GSH	Mice, Caco2, HIEC6	Promote bacterial colonization	(Dar et al., 2022)
*S. aureus*	Regulate the levels of Fe^2+^ and LPO	RAW264.7	Inhibit bacterial infection	(Ma et al., 2022b)
	Regulate the SIRT1/GPX4 axis	Mice	Inhibit inflammation	(Zhao et al., 2023a)
	Induce excessive iron accumulation and impaired antioxidant production	Mice	Promote the occurrence of mouse mastitis inflammation	(Zhao et al., 2023b)
	luteolin can inhibit ferroptosis via activate the Nrf2/GPX4 signaling pathway	Mice	Inhibit inflammation	(Gao et al., 2024b)
*E. coli*	Increase Fe^2+^ and LPO levels	RAW264.7	Inhibit bacterial infection	(Ma et al., 2022b)
	Lipid peroxidation and DNA damage	/	Promote bacteria death	(Jing et al., 2024)
*S. pullorum*	Increase Fe^2+^ and LPO levels	RAW264.7	Inhibit bacterial infection	(Ma et al., 2022b)
	Reduce GSH and GPX4, and increase ROS and MDA	U87	Promote cell death	(Chen et al., 2023a)
*Brucella*	Regulate the levels of GPX4/GSH	RAW264.7	Promote bacterial infection	(Hu et al., 2023b)
*S. cerevisiae*	Increased ROS and PUFA	/	Unclear	(Kwun and Lee, 2023)
*H. pylori*	Modulate immune activity via SOCS1	/	Regulate immune activity	(Yan et al., 2023)
*L. monocytogenes*	Regulate metabolic markers of ferroptosis	Intestinal organoids, mice	Unclear	(Zhou et al., 2022)
*F. nucleatum*	Modulate ferroptosis signaling pathway	PDLSC	Promote host inflammation response	(Wang et al., 2023b)
*E. piscicida*	Induce a c-di-GMP-mediated non-canonical ferroptosis mode	Hela	Promote cell death	(Wen et al., 2022)
*C. perfringens*	Via GPX4/FSP1/xCT and NCOA4/TF/TFR axis	THP-1	Induce macrophage injury	(Zhang et al., 2024b)
*P. gingivalis*	Via NF-κB signaling pathway	L-02	Triggers inflammation	(Yao et al., 2024)

M. tuberculosis Mycobacterium tuberculosis, P. aeruginosa Pseudomonas aeruginosa, S. aureus Staphylococcus aureus, E. coli Escherichia coli, S. pullorum Salmonella pullorum, S. cerevisiae Saccharomyces cerevisiae, H. pylori Helicobacter pylori, L. monocytogenes Listeria monocytogenes, F. nucleatum Fusobacterium nucleatum, E. piscicida Edwardsiella piscicida, C. perfringens Clostridium perfringens, P. gingivalis Porphyromonas gingivalis.

### 4.3 Ferroptosis in fungal and parasitic infections

Research on the relationship between fungi and ferroptosis is still in its early stages, but recent studies suggest its physiological relevance in fungal pathogens (**Table 3**). Previous fungal research primarily focused on intracellular iron ion balance and redox regulation, predating the concept of ferroptosis. Despite this, current findings indicate that ferroptosis significantly influences fungal infection pathways (**Figure 7**). Specifically, *Aspergillus melanogenum* (*A. melanogenum*) induces ferroptosis in murine macrophages (RAW 264.7) via a novel polysaccharide (AUM-1) that downregulates regulates GPX4 and promotes lipid peroxidation (Lin et al., 2022). In a distinct fungal context, *Magnaporthe oryzae* (*M. oryzae*) induces iron and ROS accumulation through the NADP-dependent malic enzyme 2(NADP-ME2) pathway, which triggers ferroptosis (Dangol et al., 2019). Together, these findings suggest that ferroptosis in fungi provides new insights for understanding pathogenic mechanisms and developing therapeutic agents targeting ferroptosis for fungal diseases.

**Table 3:** List of the mechanisms between the fungi or parasites and ferroptosis.

Pathogens	Mechanisms	Experimental models	Outcomes	Reference
*C. albicans*	Trigger innate immunity mode to induce ferroptosis	Mice	Induce inflammation response	(Lilly et al., 2023)
	Activation of ferroptosis and type I interferon pathway	Vaginal epithelial cells	Unclear	(Sala et al., 2023)
	Ferroptosis occur by directly exposed to FeSO4	Mice	Promote pathogen death	(Mo et al., 2024)
*P. citrinum*	Decrease LPO and HMOX1 expression	A735, CCRF-CEM	Inhibit cell death	(Yu et al., 2023)
*A. versicolor*	Inhibit ferroptosis induced by Erastin/RSL3	HT1080	Inhibit cell death	(Cui et al., 2023)
*A. melanogenum*	Regulate GPX4 and LPO	RAW 264.7	Promote cell death	(Lin et al., 2022)
*A. flavus*	Induce ferroptosis via NOXs	Conidia	Inhibit pathogen infection	(Yao et al., 2021)
*S. pombe*	The loss of the PPR gene Ppr2 leads to imbalances in iron homeostasis and redox	*S. pombe* cells	Promote pathogen death	(Liu et al., 2022)
*S. cerevisiae*	Increase ROS and PUFA	/	Unclear	(Kwun and Lee, 2023)
*M. oryzae*	Induce ROS by NAD/ME2 pathway	/	Promote pathogen infection	(Dangol et al., 2019)
*C. neoformans*	Iron overload induces neurologic damage in mice	mice	Induce damage from meningeal inflammation in mice	(Barluzzi et al., 2002)
*M. leprae*	Induce CYBB-mediated macrophage ferroptosis	Macrophage	Promote immune escape and growth of pathogenic bacteria	(Wang et al., 2024)
Malaria	By blocking the SLC7A11-GPX4 pathway	Hepa1-6, 293FT	Inhibit pathogen infection	(Kain et al., 2020)
	Activated CD8+ T cells	Primary cortical neuron	Promote damage to the nervous system	(Liang et al., 2022)
	Regulate HO-1	Mice	Induce adverse outcomes in pregnant mice	(Cariaco et al., 2022)
*T.gondii*	DHA combined with RSL3 significantly enhanced the efficacy against *T.gondii*	Vero	Inhibit pathogen infection	(Huang et al., 2022)
	Activate ferroptosis-related signaling pathways	Mice	Promote inflammation response	(Wang et al., 2023a)
*Trypanosomes*	Induce LPO	/	Promote pathogen death	(Bogacz and Krauth-Siegel, 2018)

C. albicans Candida albicans, P. citrinum Penicillium citrinum, A. versicolor Aspergillus versicolor, A. melanogenum Aspergillus melanogenum, Aspergillus flavus, S. pombe Schizosaccharomyces pombe, S. cerevisiae Saccharomyces cerevisiae, M. oryzae Magnaporthe oryzae, C. neoformans Cryptococcus neoformans, M. leprae Mycobacterium leprae, T.gondii Toxoplasma gondii.

Paralleling the emerging role of ferroptosis in fungal infections, ferroptosis has also been implicated in parasitic infections, where parasites exploit host iron metabolism and lipid peroxidation pathways to modulate cell death. Similarly to fungal research, this remains a burgeoning field. Parasites can influence ferroptosis during infection by affecting host iron metabolism and ferroptosis regulatory factors. Current studies have confirmed the involvement of ferroptosis in malaria, *Toxoplasma gondii* (*T. gondii*), and *Trypanosome* infections (**Figure 7**). Among these, *Plasmodium* spp. generate ROS and lipid peroxidation by inhibiting the SLC7A11/GPX4 pathway, thereby activating ferroptosis to clear hepatic-stage malaria (Kain et al., 2020). In a related manner, *T. gondii* infection activates ferroptosis in mice, causing pathological damage (Huang et al., 2022; Wang et al., 2023a). Collectively, while further investigation is warranted, these studies advance our understanding of parasitic infection mechanisms and highlight ferroptosis as a promising therapeutic target. **Table 3** summarizes the roles of ferroptosis in fungal and parasitic infections, highlighting distinct regulatory mechanisms across different pathogens.

## 5 The therapeutic potential of ferroptosis

### 5.1 Viral infections

Viral infections remain a major challenge to global health and economic stability. While antibody therapies and vaccines are central to current antiviral approaches, their effectiveness can be compromised by factors such as limited coverage, waning efficacy, and the potential for antibody-dependent enhancement, particularly amid ongoing viral mutations (Merad and Martin, 2020; Xu et al., 2024). Consequently, the search for new therapeutic targets has become increasingly urgent.

Burton et al. revealed that EBV-infected Burkitt-like cells generate significantly more lipid ROS than lymphoblastoid cell lines (LCL) when exposed to the ferroptosis inducer buthionine sulfoximine, highlighting their increased vulnerability to ferroptosis (Burton et al., 2022). These results indicate that promoting ferroptosis could be a viable therapeutic approach for certain EBV-related tumors. Additionally, ferroptosis inducers such as RSL3, BQR, and Erastin have been shown to activate ferroptosis and inhibit the *in vitro* replication of PEDV and ASFV (Chen et al., 2023b; Li et al., 2023c; Zhang et al., 2023a).

Modulating iron metabolism is another promising antiviral strategy. Many viral infections elevate intracellular iron levels, which support viral replication and contribute to disease severity. Iron chelators, including ICL670, 311, and DFO, have demonstrated the ability to suppress HIV-1 replication by inhibiting viral transcription and activation (Debebe et al., 2007; Salhi et al., 1998; Tabor et al., 1991). Comparable methods are also effective against large DNA viruses-such as cowpox and HSV-1-that rely on iron-dependent enzymes (Khadivjam et al., 2017; Romeo et al., 2001). By reducing cellular iron availability, chelation therapy can restrict viral proliferation and help protect cells from infection-induced damage.

In addition to iron chelation, certain ferroptosis-modulating compounds, such as antioxidants like vitamin E and butylated hydroxytoluene, have shown effectiveness against viruses including SARS-CoV-2, HSV, HIV, et al (Richards et al., 1985; Spada et al., 2002; Wang et al., 2020). Furthermore, ferrous-reactive endoperoxides, such as artemisinin and its derivatives, also display antiviral activity, likely by influencing iron metabolism and oxidative stress (Creek et al., 2007; Efferth et al., 2008; Zhou et al., 2021). Despite these advances, the exact antiviral mechanisms of many ferroptosis regulators remain inadequately understood and require further study. Indeed, research exploring the relationship between ferroptosis and various viruses, including hepatitis viruses, HIV, JEV, and SARS-CoV-2, is evolving rapidly (Carr et al., 2019; Kuo et al., 2020; Xiao et al., 2022; Yamane et al., 2022).

Nonetheless, the precise pathophysiological links between ferroptosis and specific viral infections remain incompletely defined. A deeper mechanistic understanding of these interactions will be essential for the rational design of ferroptosis-targeted antiviral therapies and the advancement of novel treatment paradigms.

However, the treatment process may encounter several challenges. First, ferroptosis-related compounds, such as Erastin, RSL3 and ML162, can lead to significant off-target effects and additional tissue damage, similar in viral infections. This lack of specificity poses a major obstacle to clinical translation, as s widespread distribution can damage healthy tissues dependent on normal iron metabolism and redox homeostasis. Therefore, targeted delivery strategies could be developed specifically for virus-infected cells. For example, virus-targeting antibodies or ligands that bind viral receptors can concentrate drugs in infected cells. An example of a targeted antiviral therapy is Lamivudine, which selectively targets viral polymerase to inhibit viral DNA synthesis by competing with deoxycytidine triphosphate (dCTP) in the treatment of HIV and HBV (De Clercq and Li, 2016). Insights gained from the development of lamivudine may inform the creation of targeted therapies that exploit ferroptosis. Furthermore, the ferroptosis inducer PF-670462 selectively targets tumor cells and reduces their resistance to ferroptosis, thereby enhancing the immune system’s ability to eliminate these tumors (Zhou et al., 2025). Drawing from these collective insights, developing ferroptosis inducers with viral selectivity remains a critical priority for clinical translation. Second, drug safety represents another major concern that requires thorough evaluation. Several compounds that can modulate ferroptosis pathways have been approved by the US Food and Drug Administration (FDA) for other indications, including DFO, deferasirox (DFX), sulfasalazine (SAS), and altretamine, for treating cancers and autoimmune diseases. However, their antiviral applications remain unexplored (Hassannia et al., 2019; Jin et al., 2020). While these agents have demonstrated acceptable safety profiles in their approved indications, their safety in the context of acute or chronic viral infections remains unclear. Potential concerns include the narrow therapeutic window, individual patient variability in iron metabolism, and possible adverse effects on host immune function and normal cellular processes dependent on iron-containing enzymes. Therefore, systematic evaluation in viral infection models is essential. In parallel, screening FDA-approved drug libraries for compounds capable of modulating ferroptosis pathways could facilitate the identification of safe and effective antiviral treatments while leveraging existing safety data to potentially reduce development timelines. Third, the generalizability of ferroptosis-related agents is constrained by the diverse clinical manifestations and progression of various viral infections. Different viruses exhibit distinct replication strategies, tissue tropisms, and interactions with host iron metabolism pathways, which may result in varying susceptibility to ferroptosis induction. This pathogen diversity necessitates the development of virus-specific or at least viral-family-specific therapeutic strategies. Given their extensive use in tumor therapy, it is crucial to prioritize research on diseases associated with EBV and HBV. The insights gained from this research could then facilitate the gradual expansion of these agents’ applications to other viral infections. However, preclinical studies must assess ferroptosis-based therapies across different viral pathogens, accounting for variations in viral load, infection stage, and host immune status.

### 5.2 Bacterial infections

Pharmacological modulation of ferroptosis has shown considerable promise in the management of bacterial infections, particularly in *M. tuberculosis* infection and sepsis. Several clinical studies have demonstrated that patients receiving vitamin E, selenium, and/or N-acetylcysteine (NAC, a glutathione precursor) as adjunctive treatments alongside standard *M. tuberculosis* antibiotic therapy exhibit an enhanced host response to treatment compared to those administered a placebo (Amaral et al., 2014; Campa et al., 2017; Ingold et al., 2018; Mahakalkar et al., 2017). Building on these clinical observations, modulating macrophage inflammatory responses and ferroptosis with various drugs has been shown to decrease the bacterial load of *M. tuberculosis* laying the groundwork for new antimicrobial agents (Amaral et al., 2019; Geng et al., 2023). The therapeutic relevance of ferroptosis modulation extends beyond *M. tuberculosis* to sepsis, a life-threatening systemic response that commonly arises from bacterial infection. Kang et al. demonstrated that GPX4 negatively regulates macrophage pyroptosis and septic lethality in mice by attenuating lipid peroxidation (Kang et al., 2018). Consistently, administration of vitamin E conferred protection to the mice from lethal sepsis (Kang et al., 2018). Of note, animals treated with NAC and deferoxamine exhibited enhanced resistance to lethal sepsis (Ritter et al., 2004; Vlahakos et al., 2012). This protective effect was attributed to increased glutathione levels and decreased availability of free iron. Taken together, these findings substantiate the therapeutic relevance of ferroptosis regulation in bacterial pathogenesis. The convergence of antioxidant supplementation, glutathione restoration, and iron chelation strategies highlights ferroptosis as a viable and multifaceted therapeutic target, warranting further clinical investigation in the treatment of bacterial infectious diseases.

### 5.3 Fungal and parasitic infections

Emerging evidence suggests that ferroptosis represents a therapeutically exploitable pathway in both fungal and parasitic infections, with distinct intervention strategies demonstrating efficacy across different pathogen models. In fungal infections, exogenous Fe^2+^ has been shown to trigger ferroptosis in *Aspergillus flavus* (*A. flavus*) spores by activating NADPH oxidases (NOXs), hindering *A. flavus* infection (Yao et al., 2021). Similarly, deferoxamine and Fer-1 successfully prevented iron-dependent ROS accumulation and lipid peroxidation, preventing cell death in rice sheaths during avirulent *M. oryzae* infection (Dangol et al., 2019). In the context of parasitic infections, treatment combined with dihydroartemisinin (DHA) and RSL3 significantly enhances resistance to Toxoplasma infection (Huang et al., 2022; Wang et al., 2023a). Additionally, Fer-1 treatment in an *in vitro* infection model resulted in a decrease in malaria parasite-associated lipid peroxides; *in vivo*, mice given Erastin before exposure to the malaria parasite had a delay of up to 2 days in the start of blood infection (Kain et al., 2020). These observations substantiate ferroptosis as a mechanistically pertinent and pharmacologically tractable target in fungal and parasitic infectious diseases.

## 6 Ferroptosis in pediatric infections

The heightened vulnerability of children to pathogen-induced ferroptosis stems from their unique developmental physiology. Children demonstrate enhanced intestinal iron absorption due to developmental upregulation of intestinal divalent metal transporter 1 (DMT1) expression during rapid growth and enhanced hepcidin sensitivity (Yanatori and Kishi, 2019). However, iron storage capacity in developing organs, particularly the liver and spleen, increases progressively with age (Lynch et al., 2018). This mismatch between iron intake and storage capacity creates a physiological vulnerability: while robust iron uptake supports accelerated hematopoiesis and tissue expansion necessary for normal growth, it simultaneously increases children’s vulnerability to pathogen-driven iron dysregulation and the subsequent activation of ferroptosis pathways.

Childhood infectious diseases exemplify this vulnerability. *M. tuberculosis* induces ferroptosis by manipulating iron homeostasis and lipid peroxidation pathways, with progressive primary pulmonary tuberculosis and tuberculous meningitis as particularly severe manifestations (Amaral et al., 2024; Amaral et al., 2014). Similarly, childhood viral infections, including EBV, SARS-CoV-2, H1N1, JEV, HAdV-7, and EV-A71, induce ferroptosis through disruption of iron homeostasis, lipid metabolism, and antioxidant defense systems (Liu et al., 2023a; Liu et al., 2025; Nguyen et al., 2022; Yang et al., 2022; Yuan et al., 2022; Zhao et al., 2024b). Among these, EBV primarily causes infectious mononucleosis in children and adolescents upon primary infection and is closely associated with pediatric malignancies such as Burkitt lymphoma (Liu et al., 2025). Notably, JEV and HAdV-7 cause severe disease in children, with JEV being a leading cause of viral encephalitis and HAdV-7 associated with severe pneumonia (Li et al., 2025; Tandale et al., 2023). Moreover, EV-A71 is the primary causative agent of hand, foot, and mouth disease (HFMD) in children (Lian et al., 2023). Beyond its role in HFMD, EV-A71 preferentially targets and replicates in human motor neurons, where it triggers neurodegeneration by inducing ferroptosis (Chooi et al., 2024). Importantly, these pathogenic stressors may produce prolonged ferroptosis effects in children because pediatric immune responses differ fundamentally from adult responses, thereby prolonging the window of pathogenic susceptibility.

Ferroptosis-targeted therapeutic strategies in children present distinctive challenges that demand careful consideration. Ferroptosis modulators must operate within an exceptionally narrow therapeutic window. Excessive iron limitation may impair normal hematopoiesis and neurological development, while inadequate dosing fails to prevent pathogen-induced ferroptosis. These constraints are further complicated by hepatorenal immaturity in children, which alters drug metabolism and clearance kinetics. Consequently, rigorous pharmacokinetic characterization and age-stratified dosing optimization are essential. Systematic research addressing these gaps will enable development of truly age-adapted therapeutic strategies that balance pathogen-specific ferroptosis susceptibility exploitation against the developmental requirements of pediatric hosts.

## 7 Convergent molecular mechanisms of pathogen-induced ferroptosis

**Figure 8:**
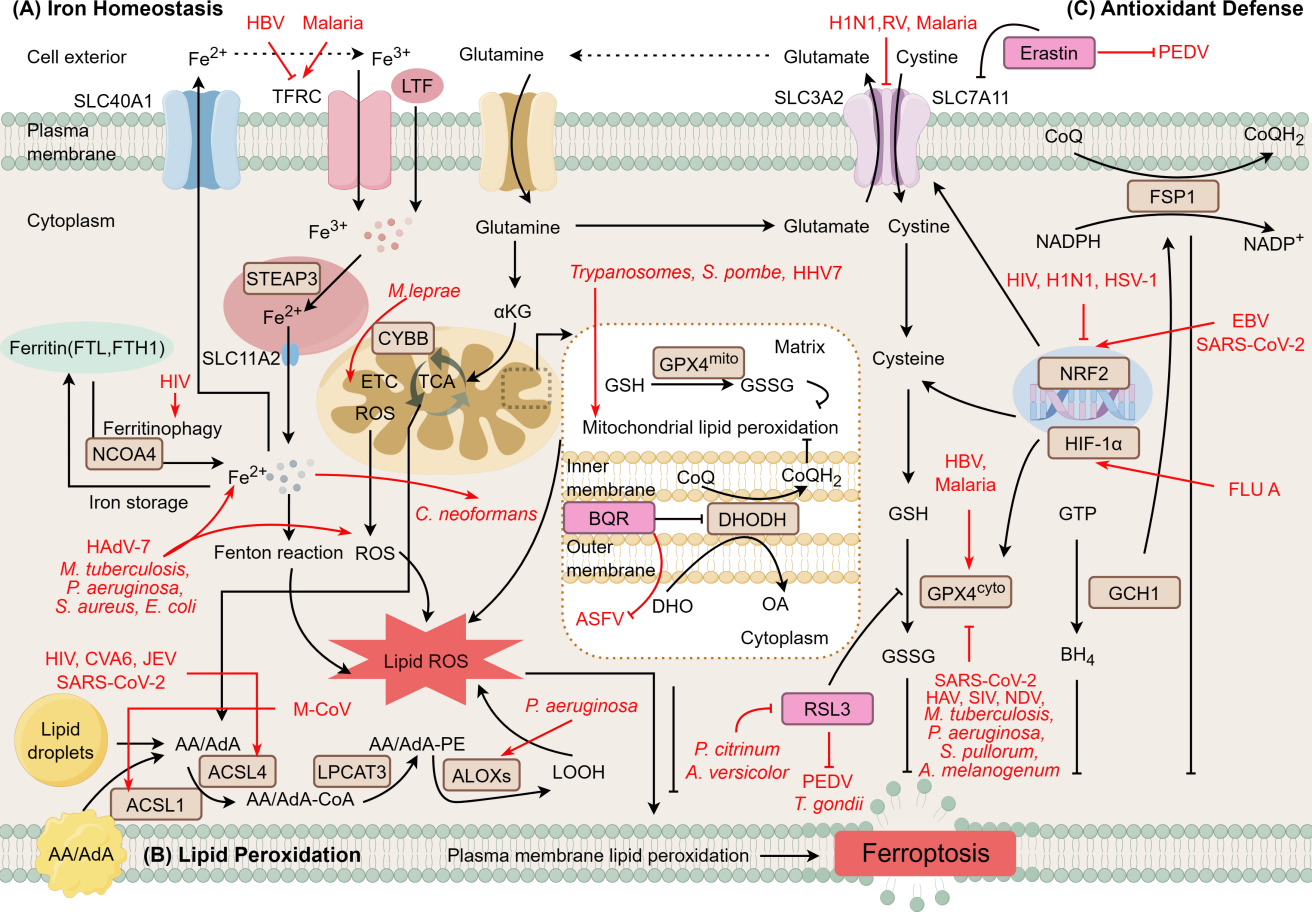
Convergent mechanisms of pathogen-induced ferroptosis. Diverse pathogens employ distinct mechanisms to drive ferroptosis by systematically targeting three critical nodes of iron homeostasis, lipid peroxidation, and antioxidant defense. **(A)** Iron homeostasis disruption:Hepatitis B virus (HBV) upregulates transferrin receptor (TFRC)-mediated Fe^2+^ influx, while Human adenovirus 7 (HAdV-7), *Mycobacterium tuberculosis* (*M. tuberculosis*), *Pseudomonas aeruginosa (P. aeruginosa*), and other bacteria promote iron accumulation through alternative pathways. **(B)** Lipid peroxidation amplification: *Trypanosomes*, *Schizosaccharomyces pombe* (*S. pombe*), and Human herpesvirus 7 (HHV-7) promote lipid peroxidation within mitochondrial membranes**;** human immunodeficiency virus (HIV), Coxsackievirus A6 (CVA6), Japanese encephalitis virus (JEV), severe acute respiratory syndrome coronavirus 2 (SARS-CoV-2), murine coronavirus (M-CoV) and *P. aeruginosa* accelerate cytoplasmic lipid peroxidation. **(C)** Antioxidant defense failure: Rhinovirus (RV) and Malaria inhibit solute carrier family 7 member 11 (SLC7A11)-mediated cystine uptake; whereas hepatitis A virus (HAV), SARS-CoV-2, *M. tuberculosis*, and *P. aeruginosa* inhibit GPX4; HIV, pandemic influenza A virus subtype H1N1 (H1N1), and Herpes simplex virus 1 (HSV-1) further attenuate antioxidant capacity by downregulating nuclear factor erythroid 2-related factor 2 (NRF2) or hypoxia-inducible factor 1-alpha (HIF-1α)-mediated transcription. Small molecule modulators including Erastin, RSL3, and BQR are shown as chemical modulators. Created by Figdraw. Arrows indicate activation (→) and inhibition (⊣).

While diverse pathogens employ distinct strategies to disrupt host cell metabolism, they remarkably converge on ferroptosis as a common cell death mechanism. Specifically, these evolutionarily distant pathogens target distinct nodes within the same ferroptosis signaling cascade, ultimately driving ferroptosis in host cells. As illustrated in **Figure 8**, all examined pathogens converge on three core molecular nodes despite their heterogeneous initial intervention strategies.

First, iron homeostasis disruption. HBV upregulates TFRC expression to promote Fe^2+^ influx, while HAdV-7, *M. tuberculosis*, and *P. aeruginosa* employ distinct mechanisms to perturb Fe^2+^ homeostasis and promote intracellular iron accumulation. The accumulated Fe^2+^ generates substantial ROS through Fenton chemistry, thereby triggering the ferroptosis cascade. Second, lipid peroxidation amplification. Trypanosomes, *S. pombe*, and HHV-7 promote mitochondrial lipid peroxidation, whereas M-CoV and *P. aeruginosa* accelerate cytoplasmic lipid peroxidation. Together, these pathogens exacerbate lipid ROS accumulation. Third, antioxidant defense failure. RV and malaria parasites inhibit SLC7A11 expression, disrupting cystine uptake and depleting GSH precursors, thereby compromising cellular antioxidant capacity. Consequently, GPX4 (both mitochondrial and cytoplasmic isoforms) loses its capacity to reduce lipid hydroperoxides. Additionally, HIV, H1N1, HSV-1, and SARS-CoV-2 further attenuate this defense pathway by downregulating NRF2 or HIF-1α expression.

The synergistic action of these three mechanisms culminates in overwhelming accumulation of lipid ROS, driving lipid peroxidation in mitochondria and cytoplasm and thus triggering ferroptosis execution. This integrative analysis reveals a conserved biological principle: despite vast evolutionary distances and heterogeneity in their infection strategies, phylogenetically diverse pathogens demonstrate remarkable convergence in their ferroptosis induction mechanisms. They systematically target three critical nodes: iron homeostasis, lipid peroxidation, and the SLC7A11-GPX4 antioxidant axis. By attacking these nodes, pathogens drive cells toward an irreversible death pathway. Consequently, modulation of these shared nodes (whether through iron chelation, GSH restoration, GPX4 stabilization, or lipid peroxidation inhibition) may offer broad-spectrum therapeutic strategies, providing a theoretical foundation for developing pan-pathogenic antimicrobial agents.

## 8 Conclusion and perspective

Ferroptosis, a regulated form of cell death, plays a significant role in the infection processes of various pathogens. It induces inflammation by accumulating lipid peroxides and iron ions, leading to the generation of reactive oxygen species. Although certain ferroptosis inhibitors can mitigate these inflammatory cascades, their therapeutic potential for pathogen infections requires further investigation. Nonetheless, studying the connection between ferroptosis and pathogen infections continues to enhance our understanding of infection processes and their underlying mechanisms.

Comparative analysis of diverse pathogens has uncovered several unifying themes in ferroptosis regulation. First, manipulation of host iron metabolism represents a common strategy employed by pathogens, achieved either through inducing iron overload or through suppressing iron-dependent ferroptosis. Second, lipid peroxidation control serves as a critical regulatory node, with pathogens modulating GPX4, GSH, and antioxidant pathways to either promote or prevent ferroptosis depending on their replication strategies. Third, the context-dependent duality of ferroptosis is evident: it can facilitate pathogen spread by eliminating immune surveillance cells or conversely restrict infection by clearing infected cells. Together, these shared mechanistic patterns suggest that ferroptosis represents a fundamental regulatory node in host-pathogen interactions, which may be commonly exploited by diverse pathogens to modulate infection outcomes.

Despite the progress made in understanding the mechanisms of ferroptosis, several significant issues remain regarding its translation into clinical therapy. First, it is essential to clarify the role of ferroptosis in infections caused by different pathogens. Such clarity will aid in comprehending its specific mechanisms across various infections and help identify new therapeutic targets. In parallel, further research on the potential role of ferroptosis in chronic infections may provide new insights for developing effective treatment strategies. Beyond mechanistic understanding, the safety and efficacy of ferroptosis-related drugs must be validated through rigorous clinical trials. Collectively, by addressing these challenges and pursuing these research directions, we can establish a solid foundation for the clinical application of ferroptosis, ultimately advancing the treatment of infectious diseases and broadening our approach to managing both acute and chronic infections.

## Glossary

ROS: reactive oxygen species
Fe^2+^: ferrous iron
PUFAs: polyunsaturated fatty acids
GPX4: glutathione peroxidase 4
ACSL4: acyl-CoA synthetase long-chain family member 4
PTGS2: prostaglandin-endoperoxide synthase 2
GSH: glutathione
SLC7A11: solute carrier family 7 member 11
RSL3: (1S,3R)-RSL3
PRDX3: peroxiredoxin 3
SARS-CoV: severe acute respiratory syndrome coronavirus
HBV: hepatitis B virus
EBV: Epstein-Barr virus
RV: rotavirus
H1N1: pandemic influenza A virus subtype H1N1
JEV: Japanese encephalitis virus
*P. aeruginosa*: *Pseudomonas aeruginosa*
*S. aureus*: *Staphylococcus aureus*
*M. tuberculosis*: *Mycobacterium tuberculosis*
FSP1: ferroptosis suppressor protein 1
AIFM2: apoptosis-inducing factor mitochondria-associated 2
NADPH: nicotinamide adenine dinucleotide phosphate
ESCRT-III: endosomal sorting complex required for transport III
DHODH: dihydroorotate dehydrogenase
CoQ10: coenzyme Q10
CoQ: coenzyme Q
CoQH₂: coenzyme QH_2_
BH_4_: tetrahydrobiopterin
GTP: guanosine triphosphate
GCH1: GTP cyclohydrolase-1
MBOAT1/MBOAT2: membrane-bound O-acyltransferase domain-containing 1/2
RBMS1: RNA binding motif single-stranded interacting protein 1
HIF-1α: hypoxia inducible factor 1 subunit alpha
SLC1A1: solute carrier family 1 member 1
LDHA: lactate dehydrogenase A
N6F11: N6-furfuryl adenine 11
HMGB1: high mobility group box 1
SEC24B: SEC24 homolog B
HBx: hepatitis B virus X protein
HAV: hepatitis A virus
HCV: hepatitis C virus
HCC: hepatocellular carcinoma
PRMT9: protein arginine methyltransferase 9
HSPA8: heat shock protein family A member 8
HSCs: hepatic stellate cells
TFRC: transferrin receptor
LF: liver fibrosis
HBsAg: hepatitis B virus surface antigen
TRIM37: tripartite motif containing 37
HIV: human immunodeficiency virus
LIP: lipase
FTH1: ferritin heavy chain
mPMs: mouse primary microglia
HAND: HIV-associated neurocognitive disorders
METH: methamphetamine
CNS: central nervous system
NRF2: NFE2 like bZIP transcription factor 2
MERS-CoV: middle east respiratory syndrome coronavirus
MARCHF1: membrane associated RING-CH-Type Finger 1
NCOA4: nuclear receptor coactivator 4
FLU A: influenza A virus
FLU B: influenza B virus
hNEPCs: human nasal epithelial progenitor cells
KEAP1: Kelch-like ECH-associated protein 1
TRIM46: tripartite motif containing 46
TAX1BP1: Tax1 binding protein 1
SQSTM1/p62: sequestosome 1
SIV: swine influenza virus
HSV-1/2: herpes simplex types 1 and 2
VZV: varicella zoster virus
HHV-6/7/8: human herpesviruses 6/7/8
NPC: nasopharyngeal carcinoma
TNFR1: tumor necrosis factor receptor 1
Cox4i2: cytochrome c oxidase subunit 4I2
MAPK: mitogen-activated protein kinase
NDV: Newcastle disease virus
HAdV-7: human adenovirus type 7
EV-A71: enterovirus A71
PRKN: E3 ubiquitin ligase parkin
YAP: Yes-associated protein
Lys90: Lysine 90
LCL: lymphoblastoid cell lines
M-CoV: murine coronavirus
CVB3: coxsackievirus B3
DENV: dengue virus
Fer-1: ferrostatin-1
DFO: deferoxamine
BQR: brequinar
PEDV: porcine epidemic diarrhea virus
ASFV: African swine fever virus
HO-1: heme oxygenase-1
BCG: Bacillus Calmette-Guérin
RNS: reactive nitrogen species
AA-PE: arachidonic acid-phosphatidylethanolamines
15-HOO-AA-PE: 15-hydroperoxy-arachidonic acid-phosphatidylethanolamines
pLoxA: lipoxygenase
HBE: human bronchial epithelial
CMA: Chaperone-mediated autophagy
NO: nitric oxide
MRSA: Methicillin-resistant strains *S. aureus*
SIRT1: sirtuin 1
*E. coli*: *Escherichia coli*
*S. pullorum*: *Salmonella pullorum*
*L. monocytogenes*: *Listeria monocytogenes*
*H. pylori*: *Helicobacter pylori*
NAC: N-acetylcysteine
*A. melanogenum*: *Aspergillus melanogenum*
*M. oryzae*: *Magnaporthe oryzae*
NADP-ME2: NADP-dependent malic enzyme 2
*T. gondii*: *Toxoplasma gondii*
dCTP: deoxycytidine triphosphate
FDA: Food and Drug Administration
DFX: deferasirox
SAS: sulfasalazine
*A. flavus*: *Aspergillus flavus*
NOXs: NADPH oxidases
DHA: Dihydroartemisinin
DMT1: divalent metal transporter 1
HFMD: hand, foot, and mouth disease.
